# 2023 update on Italian guidelines for the treatment of type 2 diabetes

**DOI:** 10.1007/s00592-023-02107-x

**Published:** 2023-05-26

**Authors:** Edoardo Mannucci, Riccardo Candido, Lina delle Monache, Marco Gallo, Andrea Giaccari, Maria Luisa Masini, Angela Mazzone, Gerardo Medea, Basilio Pintaudi, Giovanni Targher, Marina Trento, Giuseppe Turchetti, Valentina Lorenzoni, Matteo Monami, for Società Italiana di Diabetologia (SID) and Associazione Medici Diabetologi (AMD)

**Affiliations:** 1 Diabetology, Azienda Ospedaliero-Universitaria Careggi, Careggi Hospital, University of Florence, Via Delle Oblate 4, 50141 Florence, Italy; 2 Diabetology, ASUI, Trieste, Italy; 3 FAND, Milan, and FederDiabete Lazio, Rome, Italy; 4 Endocrinology and Metabolic Diseases, Hospital of Alessandria, Alessandria, Italy; 5 Endocrinology and Metabolic Diseases, Gemelli Hospital, Catholic University of Rome, Rome, Italy; 6 University of Florence, Florence, Italy; 7 San Martino Hospital, Genoa, Italy; 8 Società Italiana di Medicina Generale (SIMG), Florence, Italy; 9 Niguarda Ca’ Granda Hospital, Milan, Italy; 10 Endocrinology, Diabetology and Metabolic Diseases, University of Verona, Verona, Italy; 11 Laboratory of Clinical Pedagogy, University of Turin, Turin, Italy; 12 Scuola Superiore S. Anna, Pisa, Italy

**LISTS OF ABBREVIATIONS AND ACRONYMS**

LG: Linea Guida

AMD: Associazione Medici Ospedalieri

SID: Società Italiana di Diabetologia

PICOS: Population, Intervention, Comparison, Outcome, Study type

MNT: Medical Nutrition Therapy

NPH: Neutral Protamine Hagedorn

AMSTAR

MH-OR: Mantel–Haenzel Odds Ratio

WMD: Weighted mean difference

GRADE: Grades of Recommendation, Assessment, Development, and Evaluation

EtD: Evidence to Decision

**GUIDELINE DEVELOPMENT TEAM**

***Coordinator***: Edoardo Mannucci, diabetologist.

***Panel members***: Riccardo Candido, diabetologist; Lina delle Monache, diabetic patient; Marco Gallo^4^, diabetologist; Andrea Giaccari, diabetologist; Maria Luisa Masini, dietitian; Angela Mazzone, nurse; Gerardo Medea, general practitioner; Basilio Pintaudi, diabetologist Giovanni Targher, diabetologist; Marina Trento, pedagogist; Giuseppe Turchetti, economist.

***Evidence Review Team***: Matteo Monami, Valentina Lorenzoni

***External reviewers***: Giampaolo Fadini^1^, Antonio Nicolucci^2^, Gianluca Perseghin^3^

^1^Department of Medicine, University of Padova; ^2^Coresearch, Pescara; ^3^Metabolic Medicine, Policilinico di Monza, Bicocca University of Milan

**CONFLICTS OF INTEREST**

The assessment of interests of members of the Guideline development team is aimed at determining conflicts of interest for each question and the actions needed for their management in the process of elaboration of the Guideline. The assessment is based on the policy of the Istituto Superiore di Sanità for the management of conflicts of interest in the development of Guideline^1^. Each interest is assessed for its nature, type, relevance for the content of the Guideline, economic value, timing and duration. The assessment includes the following information which can be of help in determining the extent to which the competing interest could reasonably affect the expert’s position: type of interest; relevance for the content of the guideline; timing and duration; position of the expert in the organization (in case of institutional interests).

With respect to type of potentially competing interests, these include:

1. Economic interests, i.e., financial relationships with organizations directly producing goods or services relevant for the guideline topic. Economic interests include any monetary transaction or value related to payments for services, property shares, stock options, patents and royalties. Relevant interest can be personal, related to family members or institutional (i.e., related to the organization in which the expert works).
2. Indirect interests, such as career advancement, social position and personal beliefs.

Interests considered can be:

1. Economic interests, i.e., financial relationships with organizations involved in products or services relevant for the subject of the guideline, including any direct payment for services, property shares, stock options, and patents or copyright royalties).

Economic interests can be either:

- personal economic interest, i.e., related to a personal financial benefit;
- familial economic interest, i.e., related to the income of family members;
- institutional economic interests, i.e., related to benefits for the institution in which the subject works.

2. Intellectual interests, i.e., benefits for career advancement and social status.

Both economic and intellectual interests can be specific (i.e., directly related to the subject of the guideline) or aspecific (when they are not related to the content of the guideline).

Any reported potentially conflicting interest is classified as:

- Level 1 (minimal or not relevant): no action needed
- Level 2 (potentially relevant): this can be managed either with
  - full participation to the development of the guideline with public disclosure of the conflict of interest at the end of the recommendation related to the interest;
  - exclusion of the subject with the competing interest from the discussion of those recommendations possibly influenced by the competing interest.
- Level 3 (relevant): this can be managed with the exclusion of the subject with the competing interest from the discussion of possibly affected recommendation, or with the total exclusion of the subject with competing interest from the elaboration of the guideline.

**DECLARATION OF POTENTIAL CONFLICTS OF INTEREST**

Al members of the panel and of the evidence review team compiled annually a declaration of potential conflicts of interest, which were collectively discussed to determine their relevance. In all cases, the reported conflicts were considered minimal or irrelevant (Level 1); therefore, all components of the panel and of the evidence review team participated to the elaboration of all recommendations.

***Panel members:*** Edoardo Mannucci received fees for training activities from Mundipharma and speaking fees from Abbott, Eli Lilly e Novo Nordisk; Riccardo Candido received consulting fees from Boehringer Ingelheim, Eli Lilly, Merck, Menarini and Roche, and speaking fees from Abbott, Eli Lilly, Mundipharma, Novo Nordisk and Sanofi; Andrea Giaccarireceived consulting fees from Abbott, AstraZeneca, Boehringer Ingelheim, Eli Lilly, Merck, Mundipharma, Novo Nordisk e Sanofi, and his Institution received research grants from Amgen and AstraZeneca; Gerardo Medea received consulting fees from AstraZeneca and Grunenthal; Basilio Pintaudi received consulting and/or speaking fees from Eli Lilly e Novo Nordisk; Giovanni Targher received consulting fees from Novartis; Giuseppe Turchetti received speaking fees from Eli Lilly, and his Institution received research grants from Merck. Lina Delle Monache, Marco Gallo, Maria Luisa Masini, Angela Mazzone and Marina Trento have no interest to declare.

***Evidence review team members:*** Matteo Monami receives speaking fees from Sanofi; Valentina Lorenzoni has no interest to declare.

***External reviewers:*** Gian Paolo Fadini received research grants from Mundipharma, consulting fees from Abbott, Boehringer, Novo Nordisk and Lilly, and speaking fees from Abbott, Novo Nordisk, Sanofi, Boehringer e AstraZeneca; Gianluca Perseghin received consulting fees from AstraZeneca, Boehringer Ingelheim, Eli Lilly, Merck, Novo Nordisk, PicDare; Antonio Nicolucci received research grants from Sanofi and Novo Nordisk.

**FINANCIAL SUPPORT**

No external financial support was collected for the development of this guideline. Travel expenses for panel meeting were paid for by Società Italiana di Diabetologia. Members of Panel and Evidence Review Team did not receive any payment for their work in developing the guideline.

**AIMS**

The two main dialectological societies in Italy (SID and AMD), with the participation of other healthcare professionals involved in the care of diabetes, formulated the first joint guidelines on the treatment of type 2 diabetes in 2021^1,2^. This guideline, aimed at providing a reference for pharmacological and non-pharmacological treatment of type 2 diabetes in adults, was directed to physicians, nurses, dietitians and educators working in Diabetes specialist clinics, general practitioners, nurses and dietitian working in territorial services or private offices, and patients with diabetes.

In this first update, the guideline panel verified the need to modify, update, add or remove clinical questions, and the opportunity of modifying the outcomes of interest and their relative relevance. In case of changes in clinical questions and/or critical outcomes, the whole process of evidence review and development of recommendation was performed anew. In all other cases, the evidence review team reviewed and updated all systematic reviews (using the same search strings) for each outcome of individual question previously published^1,2^, verifying whether new evidences modified the risk/benefit ratio or the overall quality of evidences to the extent of modifying the formulation of a recommendation, of its strength or of the quality of evidence.

The following areas were assessed: therapeutic goals, nutritional therapy, physical exercise, educational programs, pharmacological treatment, glucose monitoring. All the interventions considered are usually reimbursed, with some regional differences for glucose monitoring devices and nutritional therapy. The recommendations presented in this update have been formulated on the basis of available evidence, independent of current reimbursement policies, and are designed as indications for healthcare professionals in charge of diabetes treatment, primarily based on clinical needs of people with diabetes and considering the existing organization of healthcare. These recommendations apply to outpatients, either in primary care or at specialist referral.

The implementation of the Guideline will be pursued through their dissemination, performed by:

1) Scientific Societies, using their websites and official journals and organizing specific activities of continuous medical education; 2) Regional healthcare systems.

**METHODS FOR GUIDELINE DEVELOPMENT**

The present update was developed following the methods described in the Manual of the National Guideline System (http://www.snlg-iss.it) as previously reported^1,2^.

**SUMMARY OF RECOMMENDATIONS**

1. **Treatment targets**

**1.1 A target HbA1c between 49 mmol/mol (6.6%) and 58 mmol/mol (7.5%) is recommended for patients with type 2 diabetes treated with drugs capable of inducing hypoglycemia.**

Strength of the recommendation: strong. Quality of evidence: low.

**1.2.1 A target HbA1c below 53 mmol/mol (7%) is recommended for patients with type 2 diabetes treated with drugs which are not capable of inducing hypoglycemia.**

*Strength of the recommendation: strong. Quality of evidence: low.*

**1.2.2 A target HbA1c of 48 mmol/mol (6.5%) or lower is suggested for patients with type 2 diabetes treated with drugs which are not capable of inducing hypoglycemia.**

*Strength of the recommendation: weak. Quality of evidence: very low.*

2. **Nutritional therapy**

**2.1 Structured Medical Nutrition Therapy is suggested for the treatment of type 2 diabetes.**

*Strength of the recommendation: weak. Quality of evidence: low.*

**2.2 We suggest a balanced (Mediterranean) diet, rather than a low-carbohydrate diet, for the treatment of type 2 diabetes.**

*Strength of the recommendation: weak. Quality of evidence: low.*

**2.3 We suggest to prefer low- glycemic, rather than high-glycemic-index nutrients, for the treatment of type 2 diabetes.**

**NEW RECOMMENDATION** *Strength of the recommendation: weak. Quality of evidence: low.*

3. **Physical exercise**

**3.1 We suggest regular physical exercise for the treatment of type 2 diabetes.**

*Strength of the recommendation: strong. Quality of evidence: moderate.*

**3.2 We suggest to prefer a threshold of 150 min per week for aerobic training in the treatment of type 2 diabetes.**

**MODIFIED RECOMMENDATION** *Strength of the recommendation: weak. Quality of evidence: very low.*

**3.3 There is no evidence to prefer combined (aerobic and resistance) training, rather than aerobic training alone, in the treatment of type 2 diabetes.**

**MODIFIED RECOMMENDATION** *Strength of the recommendation: weak. Quality of evidence: very low.*

4. **Educational therapy**

**4.1 We suggest structured educational therapy for the treatment of type 2 diabetes.**

*Strength of the recommendation: weak. Quality of evidence: very low.*

**4.2 We suggest grouped-based educational programs, rather than individual, for the treatment of type 2 diabetes.**

*Strength of the recommendation: weak. Quality of evidence: very low.*

5. **Pharmacological treatment**

**5.1 We recommend the use of metformin as a first-line long-term treatment in patients with type 2 diabetes without previous cardiovascular events and chronic renal failure. SGLT-2 inhibitors or GLP-1 receptor agonists are recommended as second-line treatments. Pioglitazone, DPP-4 inhibitors, acarbose, and insulin should be considered as third-line treatments. Sulfonylureas and glinides should not be recommended for the treatment of type 2 diabetes** (Fig. 1)

**Fig. 1:**
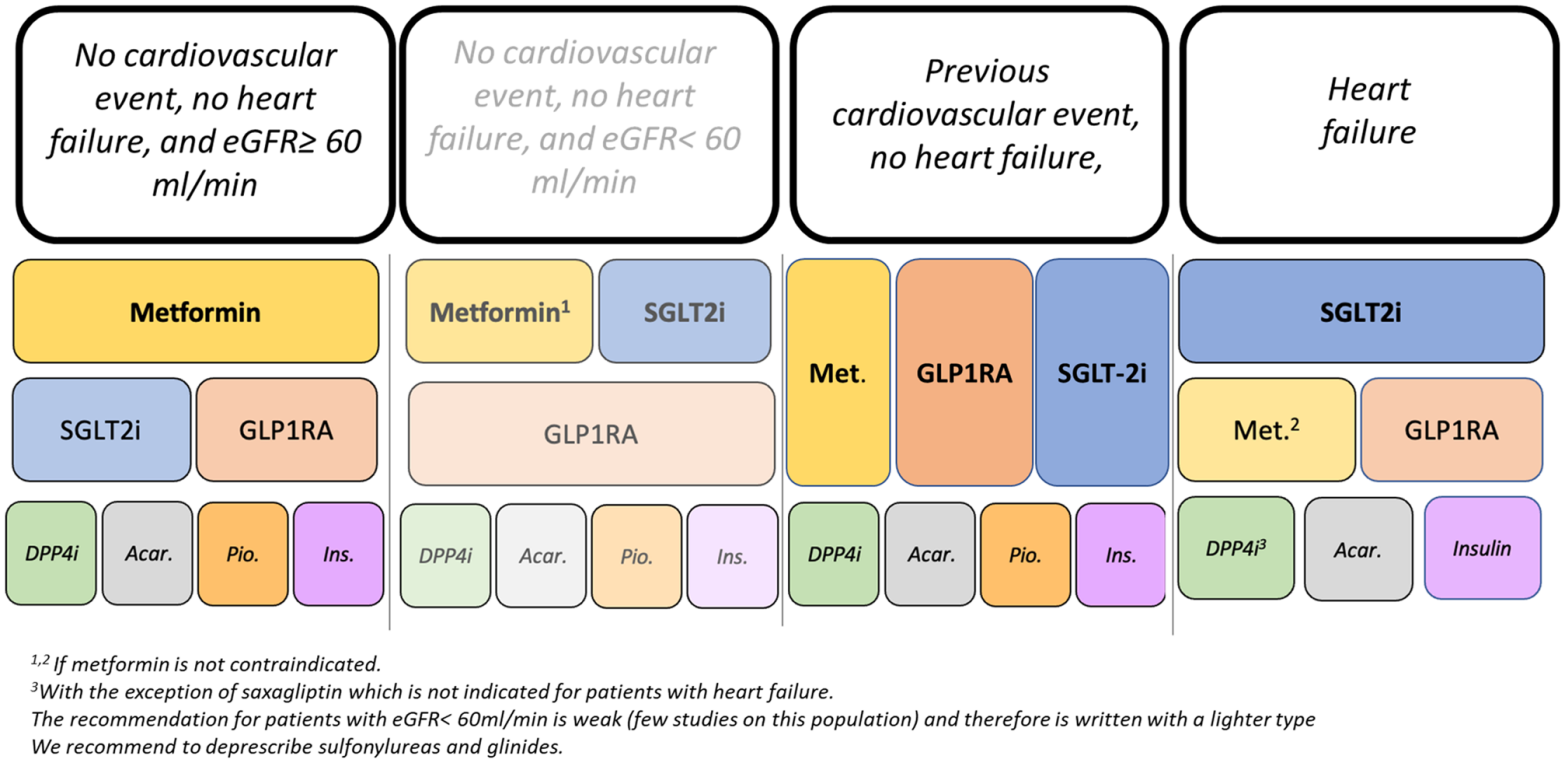
Therapeutic algorithm for the pharmacological treatment of type 2 diabetes

**MODIFIED RECOMMENDATION** *Strength of the recommendation: strong. Quality of evidence: moderate.*

**5.2. We suggest the use of metformin and SGLT-2 inhibitors as a first-line long-term treatment in patients with type 2 diabetes and eGFR < 60 ml/min, without previous cardiovascular events/heart failure. GLP-1 receptor agonists are recommended as second-line treatments. Pioglitazone, DPP-4 inhibitors, acarbose, and insulin should be considered as third-line treatments. Sulfonylureas and glinides should not be recommended for the treatment of type 2 diabetes** (Fig. 1)**.**

**NEW RECOMMENDATION** *Strength of the recommendation: weak. Quality of evidence: very low.*

**5.3. We recommend the use of metformin, SGLT-2 inhibitors, or GLP-1 receptor agonists as first-line long-term treatment in patients with type 2 diabetes with previous cardiovascular events and without heart failure. DPP-4 inhibitors, pioglitazone, acarbose, and insulin should be considered as second-line treatments. Sulfonylureas and glinides should not be recommended for the treatment of type 2 diabetes** (Fig. 1)**.**

**MODIFIED RECOMMENDATION** *Strength of the recommendation: strong. Quality of evidence: moderate.*

**5.4. We recommend the use of metformin, SGLT-2 inhibitors, or GLP-1 receptor agonists as first-line long-term treatment in patients with type 2 diabetes with previous cardiovascular events and without heart failure. DPP-4 inhibitors, pioglitazone, acarbose, and insulin should be considered as second-line treatments. Sulfonylureas and glinides should not be recommended for the treatment of type 2 diabetes** (Fig. 1)**.**

**MODIFIED RECOMMENDATION** *Strength of the recommendation: strong. Quality of evidence: moderate.*

**5.5 We suggest the use of prandial insulin analogues for patients with type 2 diabetes needing treatment with prandial insulin.**

*Strength of the recommendation: weak. Quality of evidence: very low.*

**5.6 We recommend the use of long-acting basal insulin with longer, instead or shorter duration, for all patients with type 2 diabetes needing treatment with basal insulin.**

**NEW RECOMMENDATION** *Strength of the recommendation: weak. Quality of evidence: very low.*

**5.7 We suggest the use of prandial insulin analogues for patients with type 2 diabetes needing treatment with prandial insulin.**

*Strength of the recommendation: weak. Quality of evidence: very low.*

**5.8 The routine use of continuous subcutaneous insulin infusion in inadequately controlled patients with type 2 diabetes is not recommended**.

*Strength of the recommendation: weak. Quality of evidence: very low.*

6. **Glycemic monitoring**

**6.1 We suggest to structure (with a pre-defined scheme of required tests) capillary blood glucose self-monitoring in the treatment of type 2 diabetes.**

*Strength of the recommendation: weak. Quality of evidence: very low.*

**6.2 We do not suggest a continuous glucose monitoring (continuous or on demand) rather than self-monitoring blood glucose in patients with type 2 diabetes on basal-bolus insulin therapy.**

*Strength of the recommendation: weak. Quality of evidence: very low.*

**1. THERAPEUTIC TARGETS**

**1.1 HbA1c target in patients treated with drugs inducing hypoglycemia**

Question: Which is the target HbA1c in patients with type 2 diabetes who are not treated with drugs capable of inducing hypoglycemia (insulin, sulfonylureas, glinides)?

**Table:** 

Population	People with type 2 diabetes treated with hypoglycemia-inducing drugs
Intervention	Intensified glucose control
Comparison	Standard glucose control
Outcome	Diabetic complications
Setting	Outpatient

Relevant outcomes

**Table:** 

Outcome	Relevance (1–9)	Critical
Microvascular complications	9	Yes
All-cause mortality	8	Yes
Severe hypoglycemia	8	Yes
Cardiovascular complications	7	Yes
Symptoms of diabetes	2	No

**RECOMMENDATION:**

**A target HbA1c between 49 mmol/mol (6.6%) and 58 mmol/mol (7.5%) is recommended for patients with type 2 diabetes treated with drugs capable of inducing hypoglycemia.**

*Strength of the recommendation: strong. Quality of evidence: low.*

**Justification.** The panel confirmed question and outcomes of interest. No further RCT has been retrieved and therefore this recommendation remained unaltered. For further details, please see the previous version of these guidelines^1,2^.

**1.2 HbA1c target in patients treated with drugs not inducing hypoglycemia**

Question: Which is the target HbA1c in patients with type 2 diabetes who are not treated with drugs capable of inducing hypoglycemia (insulin, sulfonylureas, glinides)?

**Table:** 

Population	People with type 2 diabetes not treated with hypoglycemia-inducing drugs
Intervention	Intensified glucose control
Comparison	Standard glucose control
Outcome	Diabetic complications
Setting	Outpatient

Relevant outcomes

**Table:** 

Outcome	Relevance (1–9)	Critical
Microvascular complications	9	Yes
All-cause mortality	8	Yes
Cardiovascular complications	7	Yes
Severe hypoglycemia	2	No
Symptoms of diabetes	2	No

**RECOMMENDATION:**

**A target HbA1c below 53 mmol/mol (7%) is recommended for patients with type 2 diabetes not treated with drugs capable of inducing hypoglycemia.**

*Strength of the recommendation: strong. Quality of evidence: low.*

**Justification**. The panel confirmed question and outcomes of interest. No further RCT has been retrieved and therefore this recommendation remained unaltered. For further details, please see the previous version of these guidelines^2^.

**RECOMMENDATION (1.2):**

**A target HbA1c of 48 mmol/mol (6.5%) or lower is suggested for patients with type 2 diabetes treated with drugs that are not capable of inducing hypoglycemia.**

*Strength of the recommendation: weak. Quality of evidence: very low.*

**Justification**. The panel confirmed question and outcomes of interest. In the previous version, no randomized trials assessed the effect of reaching and maintaining HbA1c ≤ 48 mmol/mol with drugs not capable of inducing hypoglycemia. The ERT have retrieved one trial^3^ not modifying the strength and quality of this recommendation (Fig. 1–3). For further details, please see the previous version of these guidelines^1,2^.

**EVIDENCES**

This recommendation is based on results of a meta-analysis on this issue^8^, which has been updated (using the same search string) up to 20/05/2022, retrieving a further new trial^3^. For further details, please see the previous version of the present guideline^2^ and Supplementary Materials (Fig. 1–3 and Table 1).

**2. NUTRITIONAL THERAPY**

**2.1 Structured Medical Nutrition Therapy vs unstructured nutritional advice**

Question: Is Medical Nutrition Therapy (MNT, composed of nutritional assessment, diagnosis, intervention, and monitoring) preferable to simple nutritional recommendations for diabetes control in people with type 2 diabetes?

**Table:** 

Population	People with type 2 diabetes
Intervention	Structured Medical Nutrition Therapy
Comparison	Unstructured nutritional advice
Outcome	Glucose control
Setting	Outpatient

Relevant outcomes

**Table:** 

Outcome	Relevance (1–9)	Critical
Medium- and long-term HbA1c	7	Yes
Body mass index	7	Yes
Treatment adherence	6	No
Patient’s preferences	6	No
Lipid profile	5	No
Hypoglycemia	3	No
Renal function	2	No

**RECOMMENDATION:**

**Structured Medical Nutrition Therapy is suggested for the treatment of type 2 diabetes**

*Strength of the recommendation: weak. Quality of evidence: low.*

***Justification.*** The panel confirmed question and outcomes of interest. No further RCT has been retrieved and therefore this recommendation remained unaltered. For further details, please see the previous version of these guidelines^1,2^.

**EVIDENCES**

This recommendation is based on results of a meta-analysis on this issue^4^, which has been updated (using the same search string) up to 20/05/2022, retrieving no further new trials. For further details, please see the previous version of the present guideline^1,2^.

**2.2 Low-carbohydrate vs balanced (Mediterranean) diet**

Question: Are low-carbohydrate diets more effective than balanced (Mediterranean) diets for glucose control in people with type 2 diabetes?

**Table:** 

Population	People with type 2 diabetes
Intervention	Low-carbohydrate diet
Comparison	Balanced (Mediterranean) diet
Outcome	Glucose control
Setting	Outpatient

Relevant outcomes

**Table:** 

Outcome	Relevance (1–9)	Critical
Medium- and long-term HbA1c	7	Yes
Body mass index	7	Yes
Treatment adherence	6	No
Patient’s preferences	6	No
Lipid profile	5	No
Hypoglycemia	5	No
Renal function	5	No

**RECOMMENDATION:**

**We suggest a balanced (Mediterranean) diet, rather than a low-carbohydrate diet, for the treatment of type 2 diabetes.**

*Strength of the recommendation: weak. Quality of evidence: low.*

**Justification.** The panel confirmed question and outcomes of interest. No further RCT has been retrieved, and therefore this recommendation remained unaltered. For further details, please see the previous version of these guidelines^1,2^. The ERT performed a further systematic research for trial exploring the effect of the two interventions on the risk of cardiovascular events and/or mortality. No head-to-head comparison RCTs were retrieved.

**EVIDENCES**

This recommendation is based on results of a meta-analysis on this issue^5^, which has been updated (using the same search string) up to 20/05/2022, retrieving no further trials. For further details, please see the previous version of the present guideline^1,2^ and Supplementary Materials (Fig. 4).

**2.3 Low- versus high-glycemic-index nutrients**

***New question***: Are low-glycemic-index nutrients more effective than high-glycemic nutrients for glucose control in people with type 2 diabetes?

**Table:** 

Population	People with type 2 diabetes
Intervention	Low glycemic index
Comparison	High glycemic index
Outcome	Glucose control
Setting	Outpatient

Relevant outcomes

**Table:** 

Outcome	Relevance (1–9)	Critical
Medium- and long-term HbA1c	7	Yes
Body mass index	7	Yes
Treatment adherence	6	No
Patient’s preferences	6	No
Lipid profile	5	No
Hypoglycemia	5	No
Renal function	5	No

**RECOMMENDATION:**

**We suggest to prefer low- glycemic, rather than high-glycemic-index nutrients, for the treatment of type 2 diabetes.**

*Strength of the recommendation: weak. Quality of evidence: low.*

**Justification.** There are only few studies enrolling a relatively low number of patients, showing several small, but significant, beneficial effects on glucometabolic control and endpoint body weight in favor of diets using low-glycemic-index nutrients. The low quality of the evidence and several methodological flaws of the included studies limit the strength of the present recommendation. The economic resources needed to implement this recommendation are trivial; however, no economic evaluations were retrieved on this issue.

***Subgroup considerations.*** None.

***Implementation.*** The awareness of healthcare professionals of the advantages of the use of low-glycemic-index nutrients could be increased by specific educational programs.

***Assessment and monitoring.*** The monitoring of this recommendation is problematic.

***Research priorities*.** Further trials with good methodological quality, comparing high versus low glycemic index, are needed to increase the strength of this recommendation.

**ASSESSMENT**

**Table:** 

**Problem** Is the problem a priority?		
**Judgment**	**Research evidence**	**Additional considerations**
Probably yes	The glycemic index ranks a carbohydrate containing food according to the amount by which it raises blood glucose levels after it is consumed in comparison with reference food (pure glucose or white bread)^6^. Dietary approaches that target postprandial glycemic excursions through changes to carbohydrate quality and quantity of the diet might have particular advantages^6, 7^	
**Desirable Effects** How substantial are the desirable anticipated effects?		
**Judgment**	**Research evidence**	**Additional considerations**
Small	Data derived from a meta-analysis recently published^8^ HbA1c − 0.32 [− 0.45; − 0.19]% in favor of low-glycemic-index nutrients BMI − 0.38 [− 0.64; − 0.16] kg/m^2^ in favor of low-glycemic-index nutrients	
**Undesirable Effects** How substantial are the undesirable anticipated effects?		
**Judgment**	**Research evidence**	**Additional considerations**
Trivial	None^8^	
**Certainty of evidence** What is the overall certainty of the evidence of effects?		
**Judgment**	**Research evidence**	**Additional considerations**
Low	Low for HbA1c; moderate for BMI	
**Values** Is there important uncertainty about or variability in how much people value the main outcomes?		
**Judgment**	**Research evidence**	**Additional considerations**
No important uncertainty or variability	No evidence of variability or uncertainty HbA1c and BMI are already considered among critical outcomes of the treatment of type 2 diabetes by scientific societies^4−6^	
**Balance of effects** Does the balance between desirable and undesirable effects favor the intervention or the comparison?		
**Judgment**	**Research evidence**	**Additional considerations**
Probably favors the intervention	Small, but significant reduction of HbA1c and BMI in favor of diet using low-glycemic-index nutrients	
**Resources required** How large are the resource requirements (costs)?		
**Judgment**	**Research evidence**	**Additional considerations**
Trivial	No additional costs	
**Certainty of evidence of required resources** What is the certainty of the evidence of resource requirements (costs)?		
**Judgment**	**Research evidence**	**Additional considerations**
No included studies	No studies explored this issue	
**Cost-effectiveness** Does the cost-effectiveness of the intervention favor the intervention or the comparison?		
**Judgment**	**Research evidence**	**Additional considerations**
No included studies	No studies explored this issue	
**Equity** What would be the impact on health equity?		
**Judgment**	**Research evidence**	**Additional considerations**
Probably no impact	No relevant differences in costs and accessibility	
**Acceptability** Is the intervention acceptable to key stakeholders?		
**Judgment**	**Research evidence**	**Additional considerations**
Varies	The mean consumption of high glycemic index in Italy is higher than that recommended in diets using low-glycemic-index nutrients^14^	The acceptability of a low-glycemic-index diet could be problematic for patients with type 2 diabetes living in Italy due to the modifications imposed by this nutritional approach
**Feasibility** Is the intervention feasible to implement?		
**Judgment**	**Research evidence**	**Additional considerations**
Probably yes	No additional resources are required	

**EVIDENCES**

There is a recent meta-analysis on this issue, which has been updated (using the same search string) by the ERT without retrieving further trials^8^.

**GRADE EVIDENCE TABLE**

**Table:** 

Certainty assessment							No. of patients		Effect		Certainty	Importance
No. of studies	Study design	Risk of bias	Inconsistency	Indirectness	Imprecision	Other considerations	Low-glycemic-index diets	Control diets	Relative (95% CI)	Absolute (95% CI)		
**Endpoint HbA1c**												
18	Randomized trials	Not serious	Serious^a^	Not serious	Serious^b^	None	720	745	–	MD **0.32% lower** (0.45 lower to 0.19 lower)	⨁⨁◯◯ Low	Critical
**Endpoint BMI**												
20	Randomized trials	Not serious	Not serious	Not serious	Serious^b^	None	673	690	–	MD **0.38 kg/M2 lower** (0.64 lower to 0.13 lower)	⨁⨁⨁◯ Moderate	Critical

**CI:** confidence interval; **MD:** mean difference.

*Explanations.*a. I2 = 75%b. Small trials, low overall number of patients enrolled

**3. PHYSICAL EXERCISE**

**Physical exercise and type 2 diabetes**

Question: Should physical exercise be recommended for diabetes control in patients with type 2 diabetes?

**Table:** 

Population	People with type 2 diabetes
Intervention	Physical exercise
Comparison	No intervention
Outcome	Glucose control, body weight, and composition
Setting	Outpatient

Relevant outcomes

**Table:** 

Outcome	Relevance (1–9)	Critical
HbA1c	8	Yes
Body mass index	7	Yes
Fat mass	7	Yes
Patient’s preferences	6	No
Lipid profile	6	No
Hypoglycemia	6	No

**RECOMMENDATION:**

**We suggest regular physical exercise for the treatment of type 2 diabetes.**

*Strength of the recommendation: strong. Quality of evidence: moderate.*

***Justification.*** The panel confirmed question and outcomes of interest. Several new RCTs^9–18^ have been retrieved modifying the strength of this recommendation, now rated “strong”. For further details, please see the previous version of these guidelines^2^.

**EVIDENCES**

This recommendation is based on results of a meta-analysis on this issue^19^, which has been updated (using the same search string) up to 20/05/2022, retrieving further new trials. For further details, please see Supplementary Materials (Fig. 5–7 and Table 2).

**3.2 Aerobic physical exercise and duration**

Question: Which is the minimum recommended duration of aerobic physical exercise for diabetes control in patients with type 2 diabetes?

**Table:** 

Population	People with type 2 diabetes
Intervention	Physical exercise > 150 min/week
Comparison	Physical exercise ≤ 150 min/week
Outcome	Glucose control, body weight, and composition
Setting	Outpatient

Relevant outcomes

**Table:** 

Outcome	Relevance (1–9)	Critical
HbA1c	8	Yes
Body mass index	7	Yes
Fat mass	7	Yes
Patient’s preferences	6	No
Lipid profile	6	No
Hypoglycemia	6	No

**RECOMMENDATION:**

**We suggest to prefer a threshold of 150 min per week for aerobic training in the treatment of type 2 diabetes.**

*Strength of the recommendation: weak. Quality of evidence: very low.*

***Justification***. There are no studies directly comparing interventions with different goals for weekly exercise. The available evidence, derived from the indirect comparisons of trials comparing aerobic training of different duration with no exercise, is insufficient to detect either benefit or harms. Several further trials^9–18^ were retrieved for this update, without modifying the strength and quality of this recommendation. For further details, please see the previous version of these guidelines^2^.

**Assessment**

**Table:** 

**Problem** Is the problem a priority?		
**Judgment**	**Research evidence**	**Additional considerations**
Probably yes	In epidemiological studies, there is a relationship between the amount of aerobic exercise (at least 150 min/week) and health outcomes^20^. The identification of a minimum useful threshold of the duration of physical exercise needed for a therapeutic effect in type 2 diabetes is clinically relevant	
**Desirable Effects** How substantial are the desirable anticipated effects?		
**Judgment**	**Research evidence**	**Additional considerations**
Small	After updating the previous meta-analysis^19^ a significant lower fat mass (%) was observed among patients allocated to the intervention group. No differences in HbA1c, BMI	
**Undesirable Effects** How substantial are the undesirable anticipated effects?		
**Judgment**	**Research evidence**	**Additional considerations**
Trivial	No relevant risk associated with physical exercise duration was detected in available RCTs, even after updating the previous meta-analysis^30^	
**Certainty of evidence** What is the overall certainty of the evidence of effects?		
**Judgment**	**Research evidence**	**Additional considerations**
Very low	Very low for all critical outcomes	
**Values** Is there important uncertainty about or variability in how much people value the main outcomes?		
**Judgment**	**Research evidence**	**Additional considerations**
No important uncertainty or variability	No evidence of variability or uncertainty HbA1c and BMI are already considered among critical outcomes of the treatment of type 2 diabetes by scientific societies	
**Balance of effects** Does the balance between desirable and undesirable effects favor the intervention or the comparison?		
**Judgment**	**Research evidence**	**Additional considerations**
Probably favors the intervention	Small but significant effect on HbA1c	
**Resources required** How large are the resource requirements (costs)?		
**Judgment**	**Research evidence**	**Additional considerations**
Trivial	No specific evidence is available on this issue	
**Certainty of evidence of required resources** What is the certainty of the evidence of resource requirements (costs)?		
**Judgment**	**Research evidence**	**Additional considerations**
Very low	No specific evidence is available on this issue	
**Cost-effectiveness** Does the cost-effectiveness of the intervention favor the intervention or the comparison?		
**Judgment**	**Research evidence**	**Additional considerations**
Probably favors the intervention	Small advantage for HbA1c at no estimated additional cost	
**Equity** What would be the impact on health equity?		
**Judgment**	**Research evidence**	**Additional considerations**
Probably no impact	No expected differences in costs and accessibility	
**Acceptability** Is the intervention acceptable to key stakeholders?		
**Judgment**	**Research evidence**	**Additional considerations**
Probably yes	No specific evidence is available on this issue	
**Feasibility** Is the intervention feasible to implement?		
**Judgment**	**Research evidence**	**Additional considerations**
Yes	No additional costs or resources are required	

**EVIDENCES**

This recommendation is based on results of a meta-analysis on this issue^8^, which has been updated (using the same search string) up to 20/05/2022, retrieving further new trials. For further details, please see the previous version of the present guideline^1,2^ and Supplementary materials (Figs. 8–10, Table 3).

**Different modalities of physical exercise**

Question: Should combined aerobic/resistance training be preferred to aerobic training only for diabetes control in patients with type 2 diabetes?

**Table:** 

Population	People with type 2 diabetes
Intervention	Physical exercise
Comparison	Combined aerobic/resistance training
Outcome	Glucose control
Setting	Outpatient

Relevant outcomes

**Table:** 

Outcome	Relevance (1–9)	Critical
HbA1c	7	Yes
Body mass index	6	No
Fat mass	6	No
Patient’s adherence	6	No
Hypoglycemia	3	No
Lipid profile	2	No

**RECOMMENDATION:**

**There is no evidence to prefer combined (aerobic and resistance) training, rather than aerobic training alone, in the treatment of type 2 diabetes.**

*Strength of the recommendation: weak. Quality of evidence: very low.*

The preference for combined aerobic and resistance training based on the greater reduction of HbA1c reported in some trials, it is not supported by the formal meta-analysis conducted including the newer available trials retrieved after updating the previous meta-analysis^30^. The inclusion of newer trials has modified this recommendation.

**Assessment**

**Table:** 

**Problem** Is the problem a priority?		
**Judgment**	**Research evidence**	**Additional considerations**
Probably yes	Aerobic exercise at least 3 days per week was recommended by most guidelines^4−6^. Resistance exercise alone or combined aerobic and resistance exercise was recommended only by a few guidelines^36, 37^. The identification of the best modality of physical exercise could be a relevant problem for the treatment of type 2 diabetes. Different types of exercise, which have differential effects on body composition, could theoretically determine different outcomes in diabetes control^29^	
**Desirable Effects** How substantial are the desirable anticipated effects?		
**Judgment**	**Research evidence**	**Additional considerations**
Small	Improvement of: HbA1c: − 0.1% (not significant reduction in favor of combined exercise) after updating the previous meta-analysis^30^	
**Undesirable Effects** How substantial are the undesirable anticipated effects?		
**Judgment**	**Research evidence**	**Additional considerations**
Trivial	No relevant risk associated with combined physical exercise was detected after updating the previous meta-analysis^30^	A post hoc analysis of the trials conducted for the present recommendation^30^ showed that combined exercise did not negatively affect blood pressure values at endpoint (systolic and diastolic blood pressure vs. aerobic exercise: − 6.1 [− 10.0, − 2.3] mmHg and − 2.8 [− 6.3, 0.63] mmHg, respectively)
**Certainty of evidence** What is the overall certainty of the evidence of effects?		
**Judgment**	**Research evidence**	**Additional considerations**
Very low	Very low for HbA1c	
**Values** Is there important uncertainty about or variability in how much people value the main outcomes?		
**Judgment**	**Research evidence**	**Additional considerations**
No important uncertainty or variability	No evidence of variability or uncertainty HbA1c is already considered among critical outcomes of the treatment of type 2 diabetes by scientific societies^4−6^	
**Balance of effects** Does the balance between desirable and undesirable effects favor the intervention or the comparison?		
**Judgment**	**Research evidence**	**Additional considerations**
Neither favors the intervention nor comparison	Small and nonsignificant reduction of HbA1c	
**Resources required** How large are the resource requirements (costs)?		
**Judgment**	**Research evidence**	**Additional considerations**
Trivial	Similar overall expenditure between the two interventions, with a reported advantage on cost for QALY for combined training^31^	
**Certainty of evidence of required resources** What is the certainty of the evidence of resource requirements (costs)?		
**Judgment**	**Research evidence**	**Additional considerations**
Very low	No specific evidence is available on this issue^31^	
**Cost-effectiveness** Does the cost-effectiveness of the intervention favor the intervention or the comparison?		
**Judgment**	**Research evidence**	**Additional considerations**
Does not favor either the intervention or the comparison	No between-group differences for any of the critical outcomes were considered	
**Equity** What would be the impact on health equity?		
**Judgment**	**Research evidence**	**Additional considerations**
Probably no impact	No expected differences in costs and accessibility	
**Acceptability** Is the intervention acceptable to key stakeholders?		
**Judgment**	**Research evidence**	**Additional considerations**
Probably yes	No specific evidence is available on this issue	
**Feasibility** Is the intervention feasible to implement?		
**Judgment**	**Research evidence**	**Additional considerations**
Yes	No additional costs or resources are required	

**EVIDENCES**

This recommendation is based on results of a meta-analysis on this issue^8^, which has been updated (using the same search string) up to 20/05/2022, retrieving further new trials. For further details, please see the previous version of the present guideline^1,2^ and Supplementary Materials (Fig. 11 and Table 4).

**4. EDUCATIONAL THERAPY**

**4.1 Structured educational therapy**

Question: Should structured educational therapy be preferable in comparison with generic advice for diabetes control in patients with type 2 diabetes?

**Table:** 

Population	People with type 2 diabetes
Intervention	Structured educational therapy
Comparison	Non-structured educational therapy
Outcome	HbA1c, hypoglycemia, short-/medium-term adherence, quality of life
Setting	Outpatient

Relevant outcomes

**Table:** 

Outcome	Relevance (1–9)	Critical
HbA1c	8	Yes
Medium-/long-term patient’s adherence	7	Yes
Hypoglycemia	7	Yes
Quality of life	7	Yes
Body mass index	6	No

**RECOMMENDATION:**

**We suggest structured educational therapy for the treatment of type 2 diabetes.**

*Strength of the recommendation: weak. Quality of evidence: very low.*

***Justification.*** The panel confirmed question and outcomes of interest. No further RCT has been retrieved, and therefore this recommendation remained unaltered. For further details, please see the previous version of these guidelines^1^.

**EVIDENCES**

This recommendation is based on results of a meta-analysis on this issue^21^, which has been updated (using the same search string) up to 20/05/2022, retrieving no further trials. For further details, please see the previous version of the present guideline^1,2^.

**4.2 Group- and individual-based educational therapy**

Question: Should group-based educational therapy be preferable in comparison with individual therapy for diabetes control in patients with type 2 diabetes?

**Table:** 

Population	People with type 2 diabetes
Intervention	Group-based educational therapy
Comparison	Individual-based educational therapy
Outcome	HbA1c, short-/medium-term adherence, quality of life
Setting	Outpatient

Relevant outcomes

**Table:** 

Outcome	Relevance (1–9)	Critical
HbA1c	8	Yes
Medium-/long-term patient’s adherence	7	Yes
Quality of life	7	Yes
Hypoglycemia	6	No
Body mass index	6	No

**RECOMMENDATION:**

**We suggest grouped-based educational programs, rather than individual, for the treatment of type 2 diabetes.**

*Strength of the recommendation: weak. Quality of evidence: very low.*

***Justification. Justification.*** The panel confirmed question and outcomes of interest. No further RCT has been retrieved, and therefore this recommendation remained unaltered. For further details, please see the previous version of these guidelines^1^.

**EVIDENCES**

This recommendation is based on results of a meta-analysis on this issue^22^, which has been updated (using the same search string) up to 20/05/2022, retrieving no further trials. For further details, including pharmacoeconomic evaluations, please see the previous version of the present guideline^1,2^.

**5. PHARMACOLOGICAL THERAPY**

**5.1 Glucose-lowering therapy in patients with type 2 diabetes and no previous cardiovascular events or chronic renal failure**

Which glucose-lowering agents should be considered as first-, second-, and third-line therapies for glycemic control in patients with type 2 diabetes and no previous cardiovascular events or chronic renal failure?

**Table:** 

Population	People with type 2 diabetes
Intervention	Glucose-lowering therapy
Comparison	Glucose-lowering therapy
Outcome	HbA1c, hypoglycemia, medium-/long-term adherence, mortality; major cardiovascular events
Setting	Outpatient

Relevant outcomes

**Table:** 

Outcome	Relevance (1–9)	Critical
Hypoglycemia	9	Yes
All-cause mortality	8	Yes
Medium-/long-term HbA1c	8	Yes
Quality of life	8	Yes
Major cardiovascular events	7	Yes
Body mass index	7	Yes
Renal function	6	No
Albuminuria	6	No
Hospitalization for heart failure	4	No
Short-term HbA1c	3	No
Genito-urinary infection	3	No
Ketosis	2	No

**RECOMMENDATION:**

**We recommend the use of metformin as a first-line long-term treatment in patients with type 2 diabetes without previous cardiovascular events and chronic renal failure. SGLT-2 inhibitors or GLP-1 receptor agonists are recommended as second-line treatments. Pioglitazone, DPP-4 inhibitors, acarbose, and insulin should be considered as third-line treatments. Sulfonylureas and glinides should not be recommended for the treatment of type 2 diabetes.**

*Strength of the recommendation: strong. Quality of evidence: moderate.*

**Justification.** The panel has modified the question (adding a statement on chronic renal disease; see above), confirming outcomes of interest. Several further RCTs have been retrieved without modifying this recommendation which remained unaltered. For further details, please see the previous version of these guidelines^2^, a recently published meta-analysis^2^, and Supplementary materials (Figs. 12–14 and Table 5).

**Assessment**

**Table:** 

**Problem** Is the problem a priority?		
**Judgment**	**Research evidence**	**Additional considerations**
Yes	Different guidelines propose different algorithms for the pharmacological treatment of type 2 diabetes. Many guidelines recommend metformin as first-line agents, but others prefer other agents in the majority of patients^23−26^. Recommendations on second- and third-line therapies are also heterogeneous^23−26^ The preference for a drug over another depends on its safety and tolerability, as well as its efficacy. Some side effects (e.g., weight gain, hypoglycemia, and gastrointestinal effects) are common with some glucose-lowering drugs. Those adverse effects, together with the complexity and potential burdens of therapy, may affect patients’ quality of life. In addition, several drugs have been shown renal and cardiovascular and/or nefro-protective effects. All those factors should be considered when selecting a drug, or a combination of drugs, for the treatment of an individual patient	
**Desirable Effects** How substantial are the desirable anticipated effects?		
**Judgment**	**Research evidence**	**Additional considerations**
Varies	**Effects of different classes of drugs, as reported in direct comparisons**^27^ (only statistical significant results are reported): *52-week HbA1c: compared to metformin* GLP-1 RA: − 0.2% Acarbose: + 0.4% *104-week HbA1c: compared to metformin* SGLT-2i: − 0.2% Sulfonylureas: + 0.1% Insulin: + 0.4% **Overall effects of different classes on MACE** ^28^ **:** Metformina: − 40%; GLP-1 RA: − 11%; SGLT-2i: − 10% Pioglitazone: − 15% Insulino-secretagogues/SU: + 19% **Overall effects of different classes on all-cause mortality:** GLP-1 RA: − 12%; SGLT-2i: − 15%; Sulfonylureas: + 11%. Despite the increased risk of mortality did not reach statistical significance in any of the trials considered, the overall mortality (combining all the trials using a meta-analytical approach) for sulfonylureas was higher in comparison with placebo/other classes **Quality of life** GLP-1RA are associated with improved quality of life in comparison with DPP-4 inhibitors or insulin	The effects on MACE and all-cause mortality derive from RCTs performed on patients with previous cardiovascular events
**Undesirable Effects** How substantial are the undesirable anticipated effects?		
**Judgment**	**Research evidence**	**Additional considerations**
Varies	Severe hypoglycemia: Sulphonylureas increase the risk of hypoglycemia (OR: 2.7) in comparison with metformin^27^	Metformin: gastrointestinal side effects; rare cases of lactic acidosis Alpha-glucosidase inhibitors: gastrointestinal side effects Sulfonylureas: weight gain; hypoglycemia Pioglitazone: fluid retention; weight gain; heart failure; bone fracture DPP-4 inhibitors: suspected pancreatitis; rare cases of pemphigoid GLP-1RA: gastrointestinal side effects; cholelithiasis; pancreatitis SGLT-2 inhibitors: genito-urinary infections; rare keto-acidosis Insulin: hypoglycemia and weight gain
**Certainty of evidence** What is the overall certainty of the evidence of effects?		
**Judgment**	**Research evidence**	**Additional considerations**
Moderate	High for MACE (with the exception of insulin: moderate); Moderate for all the other clinical outcomes	
**Values** Is there important uncertainty about or variability in how much people value the main outcomes?		
**Judgment**	**Research evidence**	**Additional considerations**
No important uncertainty or variability	No evidence of variability or uncertainty HbA1c, body weight, severe hypoglycemia, macrovascular complications, and mortality are already considered among critical outcomes of the treatment of type 2 diabetes by scientific societies^23, 26, 29^	
**Balance of effects** Does the balance between desirable and undesirable effects favor the intervention or the comparison?		
**Judgment**	**Research evidence**	**Additional considerations**
Varies	The balance of effects favor metformin, GLP-1 RA, and SGLT-2i over other classes of drugs, whereas it is unfavorable for sulfonylureas	
**Resources required** How large are the resource requirements (costs)?		
**Judgment**	**Research evidence**	**Additional considerations**
Varies	Low for metformin, pioglitazone, sulfonylureas, acarbose Moderate for other classes, higher for GLP-1RA and insulin	Some bioequivalent molecules could reduce direct costs for the most expensive approaches (i.e., insulin and GLP-1RA)
**Certainty of evidence of required resources** What is the certainty of the evidence of resource requirements (costs)?		
**Judgment**	**Research evidence**	**Additional considerations**
High	Several good-quality studies explored this issue	
**Cost-effectiveness** Does the cost-effectiveness of the intervention favor the intervention or the comparison?		
**Judgment**	**Research evidence**	**Additional considerations**
Varies	The cost-effective evaluation depends on the form of the drug used	
**Equity** What would be the impact on health equity?		
**Judgment**	**Research evidence**	**Additional considerations**
Probably no impact	Drugs recommended in the present guideline are already considered as first- and second-line treatments for patients without previous cardiovascular events in the principal guidelines^23, 24, 26, 29^	
**Acceptability** Is the intervention acceptable to key stakeholders?		
**Judgment**	**Research evidence**	**Additional considerations**
Probably yes	No specific evidence is available on this issue	
**Feasibility** Is the intervention feasible to implement?		
**Judgment**	**Research evidence**	**Additional considerations**
Probably yes	A large part of patients with type 2 diabetes in Italy is already treated with metformin, whereas GLP-1 RA and SGLT-2i are still relatively underutilized and sulfonylureas still prescribed^23, 26, 29^	

**EVIDENCES**

There is a recent meta-analysis on this issue, which has been performed for the present update^28^. For further details, including pharmacoeconomic evaluations, please see also the previous version of this guidelines^1,2^, a recent published meta-analysis^28^, and Supplementary Materials (Figs. 12–14 and Table 5).

**5.2 Glucose-lowering therapy in patients with type 2 diabetes and chronic renal failure without previous cardiovascular events**

***New question***: Which glucose-lowering agents should be considered as first-, second-, and third-line therapies for glycemic control in patients with type 2 diabetes and chronic renal failure, without previous cardiovascular events?

**Table:** 

Population	People with type 2 diabetes
Intervention	Glucose-lowering therapy
Comparison	Glucose-lowering therapy
Outcome	HbA1c, hypoglycemia, medium-/long-term adherence, mortality; major cardiovascular events
Setting	Outpatient

Relevant outcomes.

**Table:** 

Outcome	Relevance (1–9)	Critical
Hypoglycemia	9	Yes
All-cause mortality	8	Yes
Medium-/long-term HbA1c	8	Yes
Quality of life	8	Yes
Major cardiovascular events	7	Yes
Body mass index	7	Yes
Renal function	6	No
Albuminuria	6	No
Hospitalization for heart failure	4	No
Short-term HbA1c	3	No
Genito-urinary infection	3	No
Ketosis	2	No

**RECOMMENDATION:**

**We suggest the use of metformin and SGLT-2 inhibitors as a first-line long-term treatment in patients with type 2 diabetes and eGFR < 60 ml/min, without previous cardiovascular events/heart failure. GLP-1 receptor agonists are recommended as second-line treatments. Pioglitazone, DPP-4 inhibitors, acarbose, and insulin should be considered as third-line treatments. Sulfonylureas and glinides should not be recommended for the treatment of type 2 diabetes.**

*Strength of the recommendation: weak. Quality of evidence: very low.*

**Justification.** There are relatively few randomized controlled trials exploring the efficacy and safety of glucose-lowering agents in patients with chronic renal failure. Therefore, the present recommendation derives only from indirect evidences, showing a superiority of SGLT-2 inhibitors over the other classes of drugs. GLP-1RA should be used as second-line treatment. Insulin-secretagogues and sulfonylureas have detrimental effects in these patients.

The quality of the evidence is very low.

Several good-quality pharmacoeconomic studies showed that metformin has the lowest direct costs in comparison with other classes of glucose-lowering agents; moreover, metformin and SGLT-2 inhibitors, and, to a lesser extent, GLP-1 receptor agonists have a good cost-effective ratio.

***Subgroup considerations.*** This recommendation provides more than one option for both second and third-line therapies. The choice among available options can be affected by patients' characteristics such as age, renal failure, body weight, duration of diabetes, comorbid conditions, diabetic complications, etc., or by clinical conditions (e.g., high degree of hyperglycemia) based on clinicians' Judgment.

***Implementation.*** Sulfonylureas should not be added to ongoing therapy; existing treatments with sulfonylureas should be progressively deprescribed or substitutes with other therapies irrespective of glycemic control.

The whole medical community should be made aware of this recommendation to homogenize the therapy for type 2 diabetes in line with evidence-based medicine. Continuing medical education programs are needed to implement the knowledge of physicians in this respect.

***Assessment and monitoring***. The monitoring of adherence to guidelines on the pharmacological treatment of type 2 diabetes can be implemented through the consultation of existing databases.

**Assessment**

**Table:** 

**Problem** Is the problem a priority?		
**Judgment**	**Research evidence**	**Additional considerations**
Yes	Different guidelines propose different algorithms for the pharmacological treatment of patients with type 2 diabetes and renal insufficiency^30^. However, there are relatively few randomized controlled trials exploring the efficacy and safety of glucose-lowering agents in patients with chronic renal failure	
**Desirable Effects** How substantial are the desirable anticipated effects?		
**Judgment**	**Research evidence**	**Additional considerations**
Varies	**Effects of different classes of drugs, as reported in direct comparisons**^27^ (only statistical significant results are reported): *52-week HbA1c: compared to metformin* GLP-1 RA: − 0.2% Acarbose: + 0.4% *104-week HbA1c: compared to metformin* SGLT-2i: − 0.2% Sulfonylureas: + 0.1% Insulin: + 0.4% **Overall effects of different classes on MACE** ^28^ **:** Metformina: − 48%; GLP-1 RA: − 11%; SGLT-2i: − 11% **Overall effects of different classes on all-cause mortality:** GLP-1 RA: − 11%; SGLT-2i: − 14%; Sulfonylureas: + 11%. Although the increased risk of mortality did not reach statistical significance in any of the trials considered, the overall mortality (combining all the trials using a meta-analytical approach) for sulfonylureas was higher in comparison with placebo/other classes **Quality of life** GLP-1RA are associated with improved quality of life in comparison with DPP-4 inhibitors or insulin	The effects on MACE and all-cause mortality derive from RCTs performed on patients with previous cardiovascular events
**Undesirable Effects** How substantial are the undesirable anticipated effects?		
**Judgment**	**Research evidence**	**Additional considerations**
Varies	Severe hypoglycemia: Sulphonylureas increase the risk of hypoglycemia (OR: 3.7) in comparison with metformin^27^	Metformin: gastrointestinal side effects; rare cases of lactic acidosis Alpha-glucosidase inhibitors: gastrointestinal side effects Sulfonylureas: weight gain; hypoglycemia Pioglitazone: fluid retention; weight gain; heart failure; bone fracture DPP-4 inhibitors: suspected pancreatitis; rare cases of pemphigoid GLP-1RA: gastrointestinal side effects; cholelithiasis; pancreatitis SGLT-2 inhibitors: genito-urinary infections; rare keto-acidosis Insulin: hypoglycemia and weight gain
**Certainty of evidence** What is the overall certainty of the evidence of effects?		
**Judgment**	**Research evidence**	**Additional considerations**
Low	Moderate for MACE (pioglitazone and sulfonylureas); Low for all the other clinical outcomes	
**Values** Is there important uncertainty about or variability in how much people value the main outcomes?		
**Judgment**	**Research evidence**	**Additional considerations**
No important uncertainty or variability	No evidence of variability or uncertainty HbA1c, body weight, severe hypoglycemia, macrovascular complications, and mortality are already considered among critical outcomes of the treatment of type 2 diabetes by scientific societies^23−26^	
**Balance of effects** Does the balance between desirable and undesirable effects favor the intervention or the comparison?		
**Judgment**	**Research evidence**	**Additional considerations**
Varies	The balance of effects favor metformin, GLP-1 RA, and SGLT-2i over other classes of drugs, whereas it is unfavorable for sulfonylureas	
**Resources required** How large are the resource requirements (costs)?		
**Judgment**	**Research evidence**	**Additional considerations**
Varies	Low for metformin, pioglitazone, sulfonylureas, acarbose Moderate for other classes, higher for GLP-1RA and insulin	Some bioequivalent molecules could reduce direct costs for the most expensive approaches (i.e., insulin and GLP-1RA)
**Certainty of evidence of required resources** What is the certainty of the evidence of resource requirements (costs)?		
**Judgment**	**Research evidence**	**Additional considerations**
High	Several good-quality studies explored this issue	
**Cost-effectiveness** Does the cost-effectiveness of the intervention favor the intervention or the comparison?		
**Judgment**	**Research evidence**	**Additional considerations**
Varies	The cost-effective evaluation depends on the form of the drug used	
**Equity** What would be the impact on health equity?		
**Judgment**	**Research evidence**	**Additional considerations**
Probably no impact	Drugs recommended in the present guideline are already considered as first- and second-line treatments for patients without previous cardiovascular events in the principal guidelines^23−26^	
**Acceptability** Is the intervention acceptable to key stakeholders?		
**Judgment**	**Research evidence**	**Additional considerations**
Probably yes	No specific evidence is available on this issue	
**Feasibility** Is the intervention feasible to implement?		
**Judgment**	**Research evidence**	**Additional considerations**
Probably yes	A large part of patients with type 2 diabetes in Italy is already treated with metformin, whereas GLP-1 RA and SGLT-2i are still relatively underutilized and sulfonylureas still prescribed	

**EVIDENCES**

There is a recent meta-analysis on this issue, which has been performed for the present update^28^. For further details, please see also Supplementary materials (Figs. 12–14 and Table 5).

**GRADE EVIDENCE TABLE**

**Table:** 

No. of studies	Risk of bias	Inconsistency	Indirectness	Imprecision	Other considerations	Certainty	Proportion of events		Relative effects (95% CI)	Absolute effects	
							Intervention	Control			
**Composite major adverse renal events**											
***Metformin***											
–	–	–	–	–	–	–	–	–	–	–	–
***Pioglitazone***											
–	–	–	–	–	–	–	–	–	–	–	–
***Insulin-secretagogues***											
–	–	–	–	–	–	–	–	–	–	–	–
***DPP-4i***											
23,471 (2 RCTs)	Not serious	Not serious	Not serious	Serious^b^	None	⨁⨁⨁◯ MODERATE	484/11697 (4.1%)	521/11774 (4.4%)	**OR 1.08** (0.95 to 1.22)	41 per 1.000	**3 higher per 1.000** (from 2 lower to 9 higher)
***GLP-1 RA***											
35,464 (4 RCTs)	Not serious	Not serious	Not serious	Not serious	Strong association	⨁⨁⨁⨁ HIGH	1462/17739 (8.2%)	1164/17725 (6.6%)	**OR 0.78** (0.69 to 0.87)	82 per 1.000	**17 lower per 1.000** (from 24 to 10 lower)
***SGLT-2i***											
43,871 (7 RCTs)	Not serious	Serious^a^	Not serious	Not serious	Strong association	⨁⨁⨁⨁ HIGH	749/19433 (3.9%)	631/24438 (2.6%)	**OR 0.68** (0.56 to 0.84)	39 per 1.000	**12 lower per 1.000** (from 17 to 6 lower)
***Alpha-glucosidase inhibitors***											
–	–	–	–	–	–	–	–	–	–	–	–
***Insulin***											
–	–	–	–	–	–	–	–	–	–	–	–

**CI:** confidence interval; **MD:** mean difference;***

^a^High heterogeneity; ^b^Small trials, low overall number of patients enrolled;

**Table:** 

No. of studies	Risk of bias	Inconsistency	Indirectness	Imprecision	Other considerations	Certainty	Proportion of events		Relative effects (95% CI)	Absolute effects	
							Intervention	Control			
**End-stage renal disease**											
***Metformin***											
3625 (1 RCT)	Not serious	Not serious	Not serious	VERY serious^b^	None	⨁⨁◯◯ LOW	24/3283 (0.7%)	2/342 (0.6%)	**OR 0.80** (0.19 to 3.39)	7 per 1.000	**1 lower per 1.000** (from 6 lower to17 higher**)**
***Pioglitazone***											
–	–	–	–	–	–	–	–	–	–	–	–
***Insulin-secretagogues***											
9658 (2 RCTs)	Serious^c^	Not serious	Not serious	Serious^a^	None	⨁⨁◯◯ LOW	17/5414 (0.3%)	13/4244 (0.3%)	**OR 1.34** (0.63 to 2.83)	3 per 1.000	**1 higher per 1.000** (from 1 lower to 6 higher)
***DPP-4i***											
37,360 (7 RCTs)	Not serious	Not serious	Not serious	Not serious	None	⨁⨁⨁⨁ HIGH	148/19088 (0.8%)	139/18272 (0.8%)	**OR 0.95** (0.75 to 1.20)	3 per 1.000	**3 higher per 1.000** (from 2 lower to 9 higher)
***GLP-1 RA***											
41,535 (6 RCTs)	Not serious	Not serious	Not serious	Not serious	None	⨁⨁⨁⨁ HIGH	185/20726 (0.9%)	163/20809 (0.8%)	**OR 0.82** (0.66 to 1.01)	9 per 1.000	**2 lower per 1.000** (from 3 lower to0 lower)
***SGLT-2i***											
49,875 (6 RCTs)	Not serious	Not serious	Not serious	Not serious	Very strong association	⨁⨁⨁⨁ HIGH	317/21655 (1.5%)	228/28220 (0.8%)	**OR 0.67** (0.56 to 0.80)	15 per 1.000	**5 lower per 1.000** (from 6 lower to3 lower)
***Alpha-glucosidase inhibitors***											
–	–	–	–	–	–	–	–	–	–	–	–
***Insulin***											
577 (1 RCT)	Serious^e^	Not serious	Not serious	Serious^a^	None	⨁⨁◯◯ LOW	152/383 (39.7%)	91/194 (46.9%)	OR 1.34 (0.95 to 1.90)	397 per 1.000	72 higher per 1.000 (from 12 lower to159 higher)

**CI:** confidence interval; **MD:** mean difference;

^a^High heterogeneity; ^b^Small trials, low overall number of patients enrolled.

**Table:** 

No. of studies	Risk of bias	Inconsistency	Indirectness	Imprecision	Other considerations	Certainty	Proportion of events		Relative effects (95% CI)	Absolute effects	
							Intervention	Control			
**Renal death**											
***Metformin***											
3625 (1 RCT)	Not serious	Not serious	Not serious	VERY serious^b^	none	⨁⨁◯◯ LOW	9/3283 (0.3%)	2/342 (0.6%)	**OR 2.14** (0.46 to 9.94)	3 per 1.000	**3 higher per 1.000** (from 1 lower to 24 higher)
***Pioglitazone***											
–	–	–	–	–	–	–	–	–	–	–	–
***Insulin-secretagogues***											
10,472 (3 RCTs)	Not serious^c^	Not serious	Not serious	Serious^a^	None	⨁⨁⨁◯ MODERATE	12/5820 (0.2%)	19/4652 (0.4%)	**OR 2.02** (0.97 to 4.21)	2 per 1.000	**2 higher per 1.000** (from 0 lower to 7 higher)
***DPP-4i***											
32,368 (8 RCTs)	Not serious	Not serious	Not serious	Not serious	None	⨁⨁⨁⨁ HIGH	15/16465 (0.1%)	11/15903 (0.1%)	**OR 0.87** (0.39 to 1.93)	1 per 1.000	**0 lower per 1.000** (from 1 lower to1 higher)
***GLP-1 RA***											
26,025 (4 RCTs)	Not serious	Not serious	Not serious	Serious^a^	None	⨁⨁⨁◯ MODERATA	11/12924 (0.1%)	13/13101 (0.1%)	**OR 1.19** (0.53 to 2.66)	1 per 1.000	**0 higher per 1.000** (from 0 lower to 1 higher)
***SGLT-2i***											
v	Not serious	Not serious	Not serious	Not serious	Very strong association	⨁⨁⨁⨁ HIGH	317/21655 (1.5%)	228/28220 (0.8%)	**OR 0.67** (0.56 to 0.80)	15 per 1.000	**5 lower per 1.000** (from 6 lower to3 lower)
***Alpha-glucosidase inhibitors***											
–	–	–	–	–	–	–	–	–	–	–	–
***Insulin***											
–	–	–	–	–	–	–	–	–	–	–	–

**CI:** confidence interval; **MD:** mean difference;

^a^High heterogeneity; ^b^Small trials, low overall number of patients enrolled.

**Table:** 

No. of studies	Risk of bias	Inconsistency	Indirectness	Imprecision	Other considerations	Certainty	Proportion of events		Relative effects (95% CI)	Absolute effects	
							Intervention	Control			
**Worsening albuminuria**											
***Metformin***											
–	–	–	–	–	–	–	–	–	–	–	–
***Pioglitazone***											
–	–	–	–	–	–	–	–	–	–	–	–
***Insulin-secretagogues***											
–	–	–	–	–	–	–	–	–	–	–	–
***DPP-4i***											
23,471 (2 RCTs)	Not serious	Serious^d^	Not serious	Serious^a^	Strong association	⨁⨁⨁◯ MODERATA	2125/11697 (18.2%)	1864/11774 (15.8%)	**OR 0.85** (0.76 to 0.95)	182 per 1.000	**23 lower per 1.000** (from 37 to 8 lower)
***GLP-1 RA***											
42,093 (5 RCTs)	Not serious	Serious^d^	Not serious	Not serious	None	⨁⨁⨁◯ MODERATA	1208/21057 (5.7%)	1006/21036 (4.8%)	**OR 0.81** (0.66 to 1.00)	57 per 1.000	**10 lower per 1.000** (from 19 to 0 lower)
***SGLT-2i***											
42,837 (5 RCTs)	Not serious	Serious^d^	Not serious	Not serious	VERY strong association	⨁⨁⨁⨁ HIGH	3456/18095 (19.1%)	3594/24742 (14.5%)	**OR 0.67** (0.55 to 0.80)	191 per 1.000	**54 lower per 1.000** (from 76 to 32 lower)
***Alpha-glucosidase inhibitors***											
–	–	–	–	–	–	–	–	–	–	–	–
***Insulin***											
–	–	–	–	–	–	–	–	–	–	–	–

**CI:** confidence interval; **MD:** mean difference;

^a^High heterogeneity; ^b^Small trials, low overall number of patients enrolled.

**5.3 Glucose-lowering therapy in patients with type 2 diabetes and previous cardiovascular events without heart failure**

Which glucose-lowering agents should be considered as first-, second-, and third-line therapies for glycemic control in patients with type 2 diabetes and previous cardiovascular events and without heart failure?

**Table:** 

Population	People with type 2 diabetes
Intervention	Glucose-lowering therapy
Comparison	Glucose-lowering therapy
Outcome	HbA1c, hypoglycemia, quality of life, mortality; major cardiovascular events; hospitalization for heart failure
Setting	Outpatient

Relevant outcomes

**Table:** 

Outcome	Relevance (1–9)	Critical
Major cardiovascular events	9	Yes
Hospitalization for heart failure	8	Yes
Hypoglycemia	8	Yes
All-cause mortality	9	Yes
Medium-/long-term HbA1c	7	Yes
Quality of life	7	Yes
Body mass index	5	No
Renal function	6	No
Albuminuria	4	No
Short-term HbA1c	3	No
Genito-urinary infection	3	No
Ketosis	3	No

**RECOMMENDATION:**

**We recommend the use of metformin, SGLT-2 inhibitors, or GLP-1 receptor agonists as first-line long-term treatment in patients with type 2 diabetes with previous cardiovascular events and without heart failure. DPP-4 inhibitors, pioglitazone, acarbose, and insulin should be considered as second-line treatments. Sulfonylureas and glinides should not be recommended for the treatment of type 2 diabetes.**

*Strength of the recommendation: strong. Quality of evidence: moderate.*

**Justification.** The panel has modified the question (separating patients with and without heart failure and creating two different questions), confirming outcomes of interest. Several further RCTs have been retrieved without modifying this recommendation which remained unaltered. For further details, please see the previous version of these guidelines^2^ and a recent published meta-analysis^28^ and Supplementary materials (Figs. 12–14 and Table 5).

**Assessment**

**Table:** 

**Problem** Is the problem a priority?		
**Judgment**	**Research evidence**	**Additional considerations**
Yes	Specific recommendations for patients with prior cardiovascular events are provided by some guidelines^23−26^. The absolute risk of cardiovascular events and all-cause mortality is particularly increased in patients with type 2 diabetes and established cardiovascular disease. The risk reduction observed with some classes of drugs for diabetes could therefore produce very relevant benefits in this subset of patients with diabetes	
**Desirable Effects** How substantial are the desirable anticipated effects?		
**Judgment**	**Research evidence**	**Additional considerations**
Varies	**Effects of different classes of drugs, as reported in direct comparisons**^27^ (only statistical significant results are reported): *52-week HbA1c: compared to metformin* GLP-1 RA: − 0.2% Acarbose: + 0.4% *104-week HbA1c: compared to metformin* SGLT-2i: − 0.2% Sulfonylureas: + 0.1% Insulin: + 0.4% **Overall effects of different classes on MACE**^28^.**:** Metformina: − 40%; GLP-1 RA: − 11%; SGLT-2i: − 15% Pioglitazone: − 15% SU/insulin secretagogues: + 19% **Overall effects of different classes on hospitalization for heart failure** ^28^ SGLT-2i: − 10% Pioglitazoine: + 30% **Overall effects of different classes on all-cause mortality** ^28^ **:** GLP-1 RA: − 12%; SGLT-2i: − 15%; Sulfonylureas: + 12% **Quality of life** GLP-1RA is associated with improved quality of life in comparison with DPP-4 inhibitors or insulin^28^	MACE: no trial was found for alpha-glucosidase inhibitors
**Undesirable Effects** How substantial are the undesirable anticipated effects?		
**Judgment**	**Research evidence**	**Additional considerations**
Varies	Severe hypoglycemia: Sulphonylureas increase the risk of hypoglycemia (OR: 2.7) in comparison with metformin^27^	Metformin: gastrointestinal side effects; rare cases of lactic acidosis Alpha-glucosidase inhibitors: gastrointestinal side effects Sulfonylureas: weight gain; hypoglycemia Pioglitazone: fluid retention; weight gain; heart failure; bone fracture DPP-4 inhibitors: suspected pancreatitis; rare cases of pemphigoid GLP-1RA: gastrointestinal side effects; cholelithiasis; pancreatitis SGLT-2 inhibitors: genito-urinary infections; rare keto-acidosis Insulin: hypoglycemia and weight gain
**Certainty of evidence** What is the overall certainty of the evidence of effects?		
**Judgment**	**Research evidence**	**Additional considerations**
Moderate	High for MACE (pioglitazone and sulfonylureas); Moderate for all the other clinical outcomes	
**Values** Is there important uncertainty about or variability in how much people value the main outcomes?		
**Judgment**	**Research evidence**	**Additional considerations**
No important uncertainty or variability	No evidence of variability or uncertainty HbA1c, body weight, severe hypoglycemia, macrovascular complications, and mortality are already considered among critical outcomes of the treatment of type 2 diabetes by scientific societies^23−26^	
**Balance of effects** Does the balance between desirable and undesirable effects favor the intervention or the comparison?		
**Judgment**	**Research evidence**	**Additional considerations**
Varies	The balance of effects favors metformin, GLP-1 RA and SGLT-2i over other classes of drugs, whereas it is unfavorable for sulfonylureas	
**Resources required** How large are the resource requirements (costs)?		
**Judgment**	**Research evidence**	**Additional considerations**
Varies	Low for metformin, pioglitazone, sulfonylureas, acarbose Moderate for other classes, higher for GLP-1RA and insulin	Some bioequivalent molecules could reduce direct costs for the most expensive approaches (i.e., insulin and GLP-1RA)
**Certainty of evidence of required resources** What is the certainty of the evidence of resource requirements (costs)?		
**Judgment**	**Research evidence**	**Additional considerations**
High	Several good-quality studies explored this issue	
**Cost-effectiveness** Does the cost-effectiveness of the intervention favor the intervention or the comparison?		
**Judgment**	**Research evidence**	**Additional considerations**
Varies	The cost-effective evaluation depends on the drug used; comprehensive network meta-analysis exploring the economic implication of the different approaches are lacking, if we consider the large availability of options	
**Equity** What would be the impact on health equity?		
**Judgment**	**Research evidence**	**Additional considerations**
Probably no impact	Drugs recommended in the present guideline are already considered as first- and second-line treatments for patients without previous cardiovascular events in the principal guidelines^23−26^	
**Acceptability** Is the intervention acceptable to key stakeholders?		
**Judgment**	**Research evidence**	**Additional considerations**
Probably yes	No specific evidence is available on this issue	
**Feasibility** Is the intervention feasible to implement?		
**Judgment**	**Research evidence**	**Additional considerations**
Probably yes	A large part of patients with type 2 diabetes in Italy is already treated with metformin, whereas GLP-1 RA and SGLT-2i are still relatively underutilized and sulfonylureas still prescribed, despite being less frequently than in the last years	

**EVIDENCES**

There is a recent meta-analysis on this issue, which has been performed for the present update^28^. For further details, including pharmacoeconomic evaluations, please see also the previous version of this guidelines^2^, a recent published meta-analysis^28^, and Supplementary materials (Figs. 12–14 and Table 5).

**5.4 Glucose-lowering therapy in patients with type 2 diabetes and heart failure**

Which glucose-lowering agents should be considered as first-, second-, and third-line therapies for glycemic control in patients with type 2 diabetes and heart failure?

**Table:** 

Population	People with type 2 diabetes
Intervention	Glucose-lowering therapy
Comparison	Glucose-lowering therapy
Outcome	HbA1c, hypoglycemia, quality of life, mortality; major cardiovascular events; hospitalization for heart failure
Setting	Outpatient

Relevant outcomes

**Table:** 

Outcome	Relevance (1–9)	Critical
Major cardiovascular events	9	Yes
All-cause mortality	9	Yes
Hospitalization for heart failure	8	Yes
Hypoglycemia	8	Yes
Medium-/long-term HbA1c	7	Yes
Quality of life	7	Yes
Body mass index	5	No
Renal function	6	No
Albuminuria	4	No
Short-term HbA1c	3	No
Genito-urinary infection	3	No
Ketosis	3	No

**RECOMMENDATION:**

**We recommend the use of metformin, SGLT-2 inhibitors, or GLP-1 receptor agonists as first-line long-term treatment in patients with type 2 diabetes with previous cardiovascular events and without heart failure. DPP-4 inhibitors, pioglitazone, acarbose, and insulin should be considered as second-line treatments. Sulfonylureas and glinides should not be recommended for the treatment of type 2 diabetes.**

*Strength of the recommendation: strong. Quality of evidence: moderate.*

**Justification.** The panel has modified the question (separating patients with and without heart failure and creating two different questions), confirming outcomes of interest. Several further RCT has been retrieved without modifying this recommendation which remained unaltered. For further details, please see the previous version of these guidelines^2^, a recent published meta-analysis^28^, and Supplementary materials (Figs. 12–14 and Table 5).

**Assessment**

**Table:** 

**Problem** Is the problem a priority?		
**Judgment**	**Research evidence**	**Additional considerations**
Yes	Specific recommendations for patients with prior cardiovascular events are provided by some guidelines^23−26^. The absolute risk of cardiovascular events and all-cause mortality is particularly increased in patients with type 2 diabetes and established cardiovascular disease. The risk reduction observed with some classes of drugs for diabetes could therefore produce very relevant benefits in this subset of patients with diabetes The availability of data on specific effects of some classes of drugs on the incidence of hospital admission for heart failure suggests considering separately patients with previous cardiovascular events and known heart failure	
**Desirable Effects** How substantial are the desirable anticipated effects?		
**Judgment**	**Research evidence**	**Additional considerations**
Varies	**Effects of different classes of drugs, as reported in direct comparisons**^27^ (only statistical significant results are reported): *52-week HbA1c: compared to metformin* GLP-1 RA: − 0.2% Acarbose: + 0.4% *104-week HbA1c: compared to metformin* SGLT-2i: − 0.2% Sulfonylureas: + 0.1% Insulin: + 0.4% **Overall effects of different classes on MACE** ^28^ **:** Metformina: − 40%; GLP-1 RA: − 11%; SGLT-2i: − 15% Pioglitazone: − 15% SU/insulin secretagogues: + 19% **Overall effects of different classes on hospitalization for heart failure** ^28^ SGLT-2i: − 10% Pioglitazoine: + 30% **Overall effects of different classes on all-cause mortality** ^28^ **:** GLP-1 RA: − 12%; SGLT-2i: − 15%; Sulfonylureas: + 12% **Quality of life** GLP-1RA is associated with improved quality of life in comparison with DPP-4 inhibitors or insulin^28^	MACE: no trial was found for alpha-glucosidase inhibitors
**Undesirable Effects** How substantial are the undesirable anticipated effects?		
**Judgment**	**Research evidence**	**Additional considerations**
Varies	Severe hypoglycemia: Sulphonylureas increase the risk of hypoglycemia (OR: 2.7) in comparison with metformin^27^	Metformin: gastrointestinal side effects; rare cases of lactic acidosis Alpha-glucosidase inhibitors: gastrointestinal side effects Sulfonylureas: weight gain; hypoglycemia Pioglitazone: fluid retention; weight gain; heart failure; bone fracture DPP-4 inhibitors: suspected pancreatitis; rare cases of pemphigoid GLP-1RA: gastrointestinal side effects; cholelithiasis; pancreatitis SGLT-2 inhibitors: genito-urinary infections; rare keto-acidosis Insulin: hypoglycemia and weight gain
**Certainty of evidence** What is the overall certainty of the evidence of effects?		
**Judgment**	**Research evidence**	**Additional considerations**
Moderate	High for MACE (pioglitazone and sulfonylureas); Moderate for all the other clinical outcomes	
**Values** Is there important uncertainty about or variability in how much people value the main outcomes?		
**Judgment**	**Research evidence**	**Additional considerations**
No important uncertainty or variability	No evidence of variability or uncertainty HbA1c, body weight, severe hypoglycemia, macrovascular complications, and mortality are already considered among critical outcomes of the treatment of type 2 diabetes by scientific societies^23−26^	
**Balance of effects** Does the balance between desirable and undesirable effects favor the intervention or the comparison?		
**Judgment**	**Research evidence**	**Additional considerations**
Varies	The balance of effects favors metformin, GLP-1 RA and SGLT-2i over other classes of drugs, whereas it is unfavorable for sulfonylureas	
**Resources required** How large are the resource requirements (costs)?		
**Judgment**	**Research evidence**	**Additional considerations**
Varies	Low for metformin, pioglitazone, sulfonylureas, acarbose Moderate for other classes, higher for GLP-1RA and insulin	Some bioequivalent molecules could reduce direct costs for the most expensive approaches (i.e., insulin and GLP-1RA)
**Certainty of evidence of required resources** What is the certainty of the evidence of resource requirements (costs)?		
**Judgment**	**Research evidence**	**Additional considerations**
High	Several good-quality studies explored this issue	
**Cost-effectiveness** Does the cost-effectiveness of the intervention favor the intervention or the comparison?		
**Judgment**	**Research evidence**	**Additional considerations**
Varies	The cost-effective evaluation depends on the drug used; comprehensive network meta-analysis exploring the economic implication of the different approaches are lacking, if we consider the large availability of options	
**Equity** What would be the impact on health equity?		
**Judgment**	**Research evidence**	**Additional considerations**
Probably no impact	Drugs recommended in the present guideline are already considered as first-and second-line treatments for patients without previous cardiovascular events in the principal guidelines^23−26^	
**Acceptability** Is the intervention acceptable to key stakeholders?		
**Judgment**	**Research evidence**	**Additional considerations**
Probably yes	No specific evidence is available on this issue	
**Feasibility** Is the intervention feasible to implement?		
**Judgment**	**Research evidence**	**Additional considerations**
Probably yes	A large part of patients with type 2 diabetes in Italy is already treated with metformin, whereas GLP-1 RA and SGLT-2i are still relatively underutilized and sulfonylureas still prescribed, despite being less frequently than in the last years	

**EVIDENCES**

There is a recent meta-analysis on this issue; which has been performed for the present update^28^. For further details, including pharmacoeconomic evaluations, please see also the previous version of this guidelines^2^, a recent published meta-analysis^28^, and Supplementary materials (Figs. 12–14 and Table 5).

**5.5 Treatment with basal insulin**

Question: Should basal insulin analogues be preferred to NPH insulin in insulin-treated patients with type 2 diabetes?

**Table:** 

*Population*	People with type 2 diabetes
*Intervention*	Basal insulin analogues
*Comparison*	NPH insulin
*Outcome*	Hypoglycemia
*Setting*	Outpatient

Relevant outcomes

**Table:** 

Outcome	Relevance (1–9)	Critical
Hypoglycemia	8	Yes
Quality of life	6	No
HbA1c	2	No
Body mass index	2	No
Ketosis	2	No

**RECOMMENDATION:**

**We recommend the use of basal insulin analogues, instead of NPH, for all patients with type 2 diabetes needing treatment with basal insulin.**

*Strength of the recommendation: strong. Quality of evidence: very low.*

**Justification.** The panel confirmed question and outcomes of interest. No further RCT has been retrieved and therefore this recommendation remained unaltered. For further details, please see the previous version of these guidelines^2^.

**EVIDENCES**

This recommendation is based on results of a meta-analysis^31^ on this issue, which has been updated (using the same search string) up to 01/03/2022, retrieving no further trials. For further details, please see the previous version of the present guideline^1,2^.

**5.6 Choice of long-acting basal insulin**

Question: Should long-acting basal insulin with longer duration (glargine U300 and degludec) be preferred to long-acting basal insulin with shorter duration (detemir and glargine U100) in patients with type 2 diabetes needing treatment with basal insulin?

**Table:** 

Population	People with type 2 diabetes
Intervention	Long-acting basal insulin with longer duration
Comparison	Long-acting basal insulin with shorter duration
Outcome	Hypoglycemia
Setting	Outpatient

Relevant outcomes

**Table:** 

Outcome	Relevance (1–9)	Critical
Hypoglycemia	8	Yes
Quality of life	6	No
HbA1c	2	No
Body mass index	2	No
Ketosis	2	No

**RECOMMENDATION:**

**We recommend the use of long-acting basal insulin with longer, instead or shorter, duration, for all patients with type 2 diabetes needing treatment with basal insulin.**

*Strength of the recommendation: strong. Quality of evidence: very low.*

**Justification**

There are several RCT showing that the use of long-acting basal insulin with longer duration of action is associated with a lower hypoglycemic risk and lower weight gain. The quality of the evidence is moderate due to some methodological flaws of the included trials (open-label studies) and high heterogeneity for some critical outcomes.

Pharmacoeconomic studies showed that direct costs of drugs are generally increased with newer formulations despite the cost-effectiveness ratio generally suggest good value for money because of the implication in terms of both QALY and the effects on the risk of events, weight gain etc.; the availability of biosimilars contains the cost of out-of-patent insulin analogues.

**Assessment**

**Table:** 

**Problem** Is the problem a priority?		
**Judgment**	**Research evidence**	**Additional considerations**
Yes	Hypoglycemia has a major impact on quality of life of insulin-treated patients^32^, and it represents a major obstacle for attaining desired glycemic goals Available data suggest that different long-acting insulin formulations are associated with different risk of hypoglycemia in type 2 diabetes^33, 34^	
**Desirable Effects** How substantial are the desirable anticipated effects?		
**Judgment**	**Research evidence**	**Additional considerations**
Large	**Effects of long-acting basal insulin analogues with longer vs shorter duration** Total hypoglycemia: -32% Nocturnal hypoglycemia: -31% No significant effect on severe hypoglycemia	
**Undesirable Effects** How substantial are the undesirable anticipated effects?		
**Judgment**	**Research evidence**	**Additional considerations**
Trivial	No relevant increase of any adverse event reported in clinical trials for the intervention vs comparator	
**Certainty of evidence** What is the overall certainty of the evidence of effects?		
**Judgment**	**Research evidence**	**Additional considerations**
Low	Low for total hypoglycemia; moderate for the other critical outcomes	
**Values** Is there important uncertainty about or variability in how much people value the main outcomes?		
**Judgment**	**Research evidence**	**Additional considerations**
No important uncertainty or variability	No expected uncertainty or variability	
**Balance of effects** Does the balance between desirable and undesirable effects favor the intervention or the comparison?		
**Judgment**	**Research evidence**	**Additional considerations**
Favors the intervention	The balance of effects of using the intervention instead of comparison is favorable for the reduction of total and nocturnal hypoglycemia	
**Resources required** How large are the resource requirements (costs)?		
**Judgment**	**Research evidence**	**Additional considerations**
Varies	Relevant direct costs^35^	The introduction of biosimilars reduced the average cost of out-of-patent long-acting insulin analogues
**Certainty of evidence of required resources** What is the certainty of the evidence of resource requirements (costs)?		
**Judgment**	**Research evidence**	**Additional considerations**
High	Several good-quality studies explored this issue	
**Cost-effectiveness** Does the cost-effectiveness of the intervention favor the intervention or the comparison?		
**Judgment**	**Research evidence**	**Additional considerations**
Probably favors the intervention	Pharmaeconomic studies showed that direct costs of drugs is generally increased with newer formulations although the cost-effectiveness ratio generally suggests good value for money because of the implication in terms of both QALY and the effects on the risk of events, weight gain etc.; the availability of biosimilars contains the cost of out-of-patent insulin analogues	The introduction of biosimilars reduced the average cost of out-of-patent long-acting insulin analogues, thus modifying the evaluation on cost-effectiveness ratio
**Equity** What would be the impact on health equity?		
**Judgment**	**Research evidence**	**Additional considerations**
Probably no impact	No impact expected (long-acting analogues with longer duration are already the standard of care)	
**Acceptability** Is the intervention acceptable to key stakeholders?		
**Judgment**	**Research evidence**	**Additional considerations**
Probably yes	Long-acting analogues with longer duration are already the standard of care	
**Feasibility** Is the intervention feasible to implement?		
**Judgment**	**Research evidence**	**Additional considerations**
Yes	Long-acting analogues with longer duration are already the standard of care	

**EVIDENCES**

This recommendation is based on results of an unpublished meta-analysis updated up to 01/05/2022 (Supplementary Materials, Figs. 15–17 and Table 6).

**5.7 Treatment with prandial insulin**

Question: Should prandial insulin analogues be preferred to human regular insulin in insulin-treated patients with type 2 diabetes?

**Table:** 

Population	People with type 2 diabetes
Intervention	Prandial insulin analogues
Comparison	Human regular insulin
Outcome	HbA1c, Hypoglycemia, Quality of Life, Patients’ preference
Setting	Outpatient

Relevant outcomes

**Table:** 

Outcome	Relevance (1–9)	Critical
Hypoglycemia	8	Yes
Quality of life	7	Yes
HbA1c	7	Yes
Patients’ preference	6	No
Body mass index	2	No
Ketosis	2	No

**RECOMMENDATION:**

**We suggest the use of prandial insulin analogues for patients with type 2 diabetes needing treatment with prandial insulin.**

*Strength of the recommendation: weak. Quality of evidence: very low.*

**Justification.** The panel confirmed question and outcomes of interest. No further RCT has been retrieved and therefore this recommendation remained unaltered. For further details, please see the previous version of these guidelines^2^.

**EVIDENCES**

This recommendation is based on results of a meta-analysis^31^ on this issue, which has been updated (using the same search string) up to 01/03/2022, retrieving no further trials. For further details, please see the previous version of the present guideline^2^.

**5.8 Treatment with continuous subcutaneous insulin infusion.**

Question: Should continuous subcutaneous insulin infusion be preferred in patients with type 2 diabetes not adequately controlled and treated with multiple daily injections?

**Table:** 

Population	People with type 2 diabetes
Intervention	Continuous subcutaneous insulin infusion
Comparison	Multiple daily injections
Outcome	HbA1c, Hypoglycemia, Quality of Life, Patients’ preference
Setting	Outpatient

Relevant outcomes

**Table:** 

Outcome	Relevance (1–9)	Critical
Hypoglycemia	8	Yes
Quality of life	8	Yes
HbA1c	8	Yes
Patients’ preference	6	No
Ketosis	4	No
Body mass index	2	No

**RECOMMENDATION:**

**The routine use of CSII in inadequately controlled patients with type 2 diabetes is not recommended.**

*Strength of the recommendation: weak. Quality of evidence: very low.*

**Justification.** The panel confirmed question and outcomes of interest. No further RCT has been retrieved, and therefore this recommendation remained unaltered. For further details, please see the previous version of these guidelines^2^.

**EVIDENCES**

This recommendation is based on results of a meta-analysis on this issue^36^, which has been updated (using the same search string) up to 01/03/2022, retrieving no further trials. For further details, please see the previous version of the present guideline^2^.

**6. Glucose monitoring**

**6.1 Structured glucose monitoring**

Question: Should structured glucose monitoring be preferable in comparison with capillary glucose monitoring for diabetes control in patients with type 2 diabetes?

**Table:** 

Population	People with type 2 diabetes
Intervention	Structured glucose monitoring
Comparison	Capillary glucose monitoring
Outcome	HbA1c
Setting	Outpatient

Relevant outcomes

**Table:** 

Outcome	Relevance (1–9)	Critical
HbA1c	7	Yes
Hypoglycemia	6	No
Patients’ preference	4	No

**RECOMMENDATION:**

**We suggest to structure (with a pre-defined scheme of required tests) capillary blood glucose self-monitoring in the treatment of type 2 diabetes.**

*Strength of the recommendation: weak. Quality of evidence: very low.*

**Justification.** The panel confirmed question and outcomes of interest. No further RCT has been retrieved, and therefore this recommendation remained unaltered. For further details, please see the previous version of these guidelines^2^.

**EVIDENCES**

This recommendation is based on results of a meta-analysis on this issue^37^, which has been updated (using the same search string) up to 01/03/2022, retrieving no further trials. For further details, please see the previous version of the present guideline^2^.

**Subcutaneous continuous glucose monitoring**

Question: Should subcutaneous continuous glucose monitoring be preferable in comparison with capillary glucose monitoring for diabetes control in patients with type 2 diabetes treated with basal-bolus insulin schemes?

**Table:** 

Population	People with type 2 diabetes
Intervention	Subcutaneous continuous glucose monitoring
Comparison	Capillary glucose monitoring
Outcome	HbA1c; Hypoglycemia; Patients’ preference
Setting	Outpatient

Relevant outcomes

**Table:** 

Outcome	Relevance (1–9)	Critical
HbA1c	8	Yes
Hypoglycemia	8	Yes
Patients’ preference	7	Yes

**RECOMMENDATION:**

**We do not suggest continuous glucose monitoring rather than self-monitoring blood glucose in patients with type 2 diabetes on basal-bolus insulin therapy.**

*Strength of the recommendation: weak. Quality of evidence: very low.*

**Justification.** The panel confirmed question and outcomes of interest. No further RCT has been retrieved, and therefore this recommendation remained unaltered. For further details, please see the previous version of these guidelines^2^.

**EVIDENCES**

This recommendation is based on results of a meta-analysis on this issue^36^, which has been updated (using the same search string) up to 01/05/2022, retrieving no further trials. For further details, please see the previous version of the present guideline^2^.

## Supplementary Information

Below is the link to the electronic supplementary material.

Supplementary file1 (PDF 1496 kb)

## References

[1] Mannucci E, Candido R, Delle Monache L (2022). Italian guidelines for the treatment of type 2 diabetes (in Eng). Nutr Metab Cardiovasc Dis: NMCD.

[2] Mannucci E, Candido R, Monache LD (2022). Italian guidelines for the treatment of type 2 diabetes (in Eng). Acta Diabetol.

[3] Jabbour SA, Frías JP, Ahmed A (2020). Efficacy and safety over 2 years of exenatide plus dapagliflozin in the DURATION-8 study: a multicenter, double-blind, phase 3, randomized controlled trial (in Eng). Diabetes Care.

[4] Møller G, Andersen HK, Snorgaard O (2017). A systematic review and meta-analysis of nutrition therapy compared with dietary advice in patients with type 2 diabetes (in Eng). Am J Clin Nutr.

[5] Silverii GA, Botarelli L, Dicembrini I (2020). Low-carbohydrate diets and type 2 diabetes treatment: a meta-analysis of randomized controlled trials (in Eng). Acta Diabetol.

[6] Foster-Powell K, Holt SH, Brand-Miller JC (2002). International table of glycemic index and glycemic load values: 2002 (in Eng). Am J Clin Nutr.

[7] Bergia RE, Giacco R, Hjorth T (2022). Differential glycemic effects of low- versus high-glycemic index mediterranean-style eating patterns in adults at risk for type 2 diabetes: the MEDGI-carb randomized controlled trial (in Eng). Nutrients.

[8] Chiavaroli L, Lee D, Ahmed A (2021). Effect of low glycaemic index or load dietary patterns on glycaemic control and cardiometabolic risk factors in diabetes: systematic review and meta-analysis of randomised controlled trials (in Eng). BMJ.

[9] Balducci S, D'Errico V, Haxhi J (2019). Effect of a behavioral intervention strategy on sustained change in physical activity and sedentary behavior in patients with type 2 diabetes: the IDES_2 randomized clinical trial (in Eng). JAMA.

[10] Balducci S, Zanuso S, Nicolucci A (2010). Effect of an intensive exercise intervention strategy on modifiable cardiovascular risk factors in subjects with type 2 diabetes mellitus: a randomized controlled trial: the Italian Diabetes and Exercise Study (IDES) (in Eng). Arch Intern Med.

[11] Chen SM, Shen FC, Chen JF, Chang WD, Chang NJ (2019). Effects of resistance exercise on glycated hemoglobin and functional performance in older patients with comorbid diabetes mellitus and knee osteoarthritis: a randomized trial (in Eng). Int J Environ Res Public Health.

[12] Ghardashi-Afousi A, Davoodi M, Hesamabadi BK (2020). Improved carotid intima-media thickness-induced high-intensity interval training associated with decreased serum levels of Dkk-1 and sclerostin in type 2 diabetes (in Eng). J Diabetes Complicat.

[13] Gholami F, Khaki R, Mirzaei B, Howatson G (2021). Resistance training improves nerve conduction and arterial stiffness in older adults with diabetic distal symmetrical polyneuropathy: a randomized controlled trial (in Eng). Exp Gerontol.

[14] Jeon YK, Kim SS, Kim JH (2020). Combined aerobic and resistance exercise training reduces circulating apolipoprotein J levels and improves insulin resistance in postmenopausal diabetic women (in Eng). Diabetes Metab J.

[15] Mohammadi A, Bijeh N, Moazzami M, Kazem K, Rahimi N (2022). Effect of exercise training on spexin level, appetite, lipid accumulation product, visceral adiposity index, and body composition in adults with type 2 diabetes (in Eng). Biol Res Nurs.

[16] Taheri S, Zaghloul H, Chagoury O (2020). Effect of intensive lifestyle intervention on bodyweight and glycaemia in early type 2 diabetes (DIADEM-I): an open-label, parallel-group, randomised controlled trial (in Eng). Lancet Diabetes Endocrinol.

[17] Vidanage D, Prathapan S, Hettiarachchi P, Wasalathanthri S (2022). Impact of aerobic exercises on taste perception for sucrose in patients with type 2 diabetes mellitus; a randomized controlled trial (in Eng). BMC Endocr Disord.

[18] Vieira ER, Cavalcanti F, Civitella F (2021). Effects of exercise and diet on body composition and physical function in older hispanics with type 2 diabetes (in Eng). Int J Environ Res Public Health.

[19] Mannucci E, Bonifazi A, Monami M (2021). Comparison between different types of exercise training in patients with type 2 diabetes mellitus: a systematic review and network metanalysis of randomized controlled trials (in Eng). Nutr Metab Cardiovasc Dis: NMCD.

[20] Moghetti P, Balducci S, Guidetti L, Mazzuca P, Rossi E, Schena F (2020). Walking for subjects with type 2 diabetes: a systematic review and joint AMD/SID/SISMES evidence-based practical guideline (in Eng). Nutr Metab Cardiovasc Dis: NMCD.

[21] Azmiardi A, Murti B, Febrinasari RP, Tamtomo DG (2021). The effect of peer support in diabetes self-management education on glycemic control in patients with type 2 diabetes: a systematic review and meta-analysis (in Eng). Epidemiol Health.

[22] Mannucci E, Giaccari A, Gallo M (2022). Self-management in patients with type 2 diabetes: Group-based versus individual education. A systematic review with meta-analysis of randomized trails (in Eng). Nutr Metab Cardiovasc Dis: NMCD.

[23] Nathan DM, Buse JB, Davidson MB (2006). Management of hyperglycemia in type 2 diabetes: a consensus algorithm for the initiation and adjustment of therapy: a consensus statement from the American Diabetes Association and the European Association for the Study of Diabetes (in Eng). Diabetes Care.

[24] Cosentino F, Grant PJ, Aboyans V (2020). 2019 ESC Guidelines on diabetes, pre-diabetes, and cardiovascular diseases developed in collaboration with the EASD (in Eng). Eur Heart J.

[25] Mannucci E, Candido R, Monache LD (2022). Italian guidelines for the treatment of type 2 diabetes (in Eng). Acta Diabetol.

[26] [NG28] Ng. https://www.nice.org.uk/guidance/ng28

[27] Mannucci E, Naletto L, Vaccaro G (2021). Efficacy and safety of glucose-lowering agents in patients with type 2 diabetes: a network meta-analysis of randomized, active comparator-controlled trials (in Eng). Nutr Metab Cardiovasc Dis: NMCD.

[28] Mannucci E, Gallo M, Giaccari A (2023). Effects of glucose-lowering agents on cardiovascular and renal outcomes in subjects with type 2 diabetes: an updated meta-analysis of randomized controlled trials with external adjudication of events (in Eng). Diabetes Obes Metab.

[29] https://www.siditalia.it/pdf/Standard%20di%20Cura%20AMD%20-%20SID%202018_protetto2.pdf. Last accessed 11 April 2021

[30] Navaneethan SD, Zoungas S, Caramori ML (2021). Diabetes management in chronic kidney disease: synopsis of the 2020 KDIGO clinical practice guideline (in Eng). Ann Intern Med.

[31] Mannucci E, Caiulo C, Naletto L, Madama G, Monami M (2021). Efficacy and safety of different basal and prandial insulin analogues for the treatment of type 2 diabetes: a network meta-analysis of randomized controlled trials (in Eng). Endocrine.

[32] Chevalier P, Vandebrouck T, De Keyzer D, Mertens A, Lamotte M (2016). Cost and co-morbidities associated with hypoglycemic inpatients in Belgium (in Eng). J Med Econ.

[33] Monami M, Marchionni N, Mannucci E (2008). Long-acting insulin analogues versus NPH human insulin in type 2 diabetes: a meta-analysis (in Eng). Diabetes Res Clin Pract.

[34] Monami M, Marchionni N, Mannucci E (2009). Long-acting insulin analogues vs. NPH human insulin in type 1 diabetes. A meta-analysis (in Eng). Diabetes Obes Metab.

[35] https://www.aifa.gov.it/documents/20142/1205984/rapporto-osmed-2019.pdf/f41e53a4-710a-7f75-4257-404647d0fe1e

[36] Dicembrini I, Mannucci E, Monami M, Pala L (2019). Impact of technology on glycaemic control in type 2 diabetes: a meta-analysis of randomized trials on continuous glucose monitoring and continuous subcutaneous insulin infusion (in Eng). Diabetes Obes Metab.

[37] Mannucci E, Antenore A, Giorgino F, Scavini M (2018). Effects of structured versus unstructured self-monitoring of blood glucose on glucose control in patients with non-insulin-treated type 2 diabetes: a meta-analysis of randomized controlled trials (in Eng). J Diabetes Sci Technol.

