# Supplementary material for: 2023 update on Italian guidelines for the treatment of type 2 diabetes
Source: Acta Diabetol. 2023 May 26;60(8):1119–51. doi: 10.1007/s00592-023-02107-x (PMC10290044; doi:10.1007/s00592-023-02107-x)
Supplement: Supplementary file 1 — Supplementary file1 (PDF 1496 kb) [file 592_2023_2107_MOESM1_ESM.pdf]

## LISTS OF ABBREVIATIONS AND ACRONYMS

MACE: Major Adverse Cardiovascular Events
MH: Mantel-Haenzel
OR: Odds Ratio
RR: Relative Risk
BMI: Body Mass Index
MD: Mean Difference (weighted)
QALY: Quality Adjusted Life Years (anni di vita aggiustati per qualità)
Min: Minute
ICUR: Incremental Cost-Utility Ratio
ICER: Incremental Cost-Effectiveness Ratio
SoC: Standard of Care
T2DM: Type 2 Diabetes Mellitus
RCT: Randomized controlled trials
GRADE: Grades of Recommendation, Assessment, Development, and Evaluation
EtD: Evidence to Decision
GLP-1 RA: Glucagon-Like Peptide-1 Receptor Agonists
SGLT-2i: Sodium-Glucose coTransporter-2 inhibitors
DPP-4i: DiPeptidyl Peptidase-4 inhibitors
SU: Sulfonylureas
CCS: Charlson Comorbidity Score
WTP: willingness to pay
LDL: Low-density Lipoprotein

## CONTENT OF THE APPENDIX

This Appendix contains detailed information only on PICO modified after updating the previous version of present guidelines and unpublished elsewhere. For unmodified PICO, please see the already published guidelines[1,2].

# RECOMMENDATION # 1: THERAPEUTIC TARGETS.

## 1.2 HbA1c target in patients treated with drugs not inducing hypoglycemia

**Figure 1** – Forest plot for trials comparing the effects of intensive glycemic control (using drugs associated with hypoglycemia) and standard care on MACE.

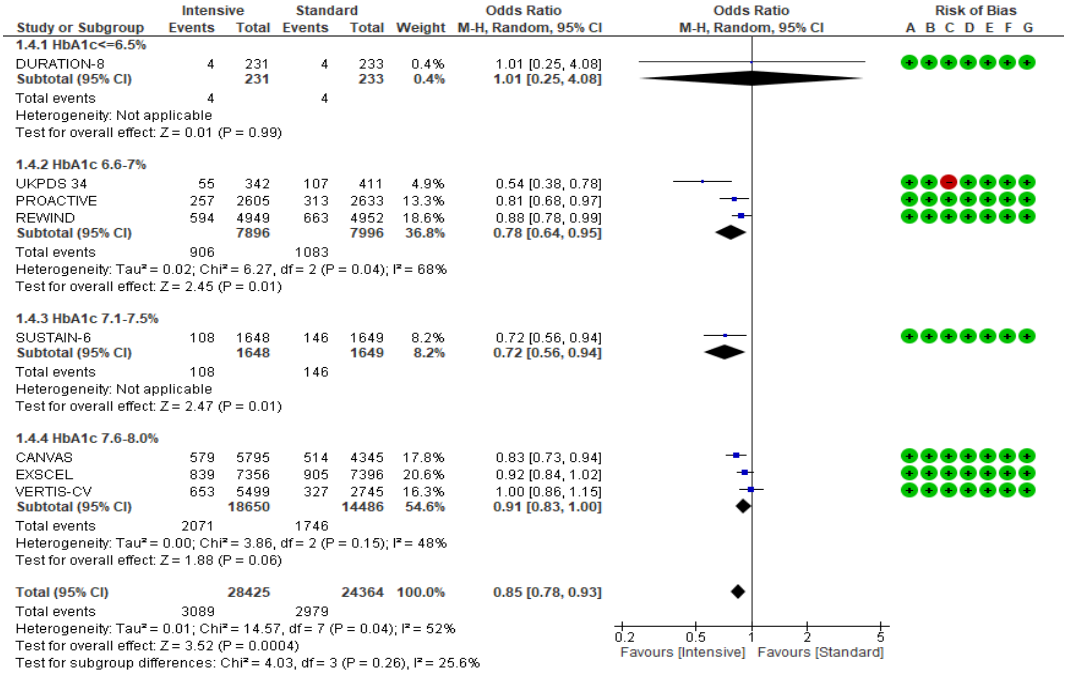


**Figure 2** – Forest plot for trials comparing the effects of intensive glycemic control (using drugs associated with hypoglycemia) and standard care on all-cause mortality.

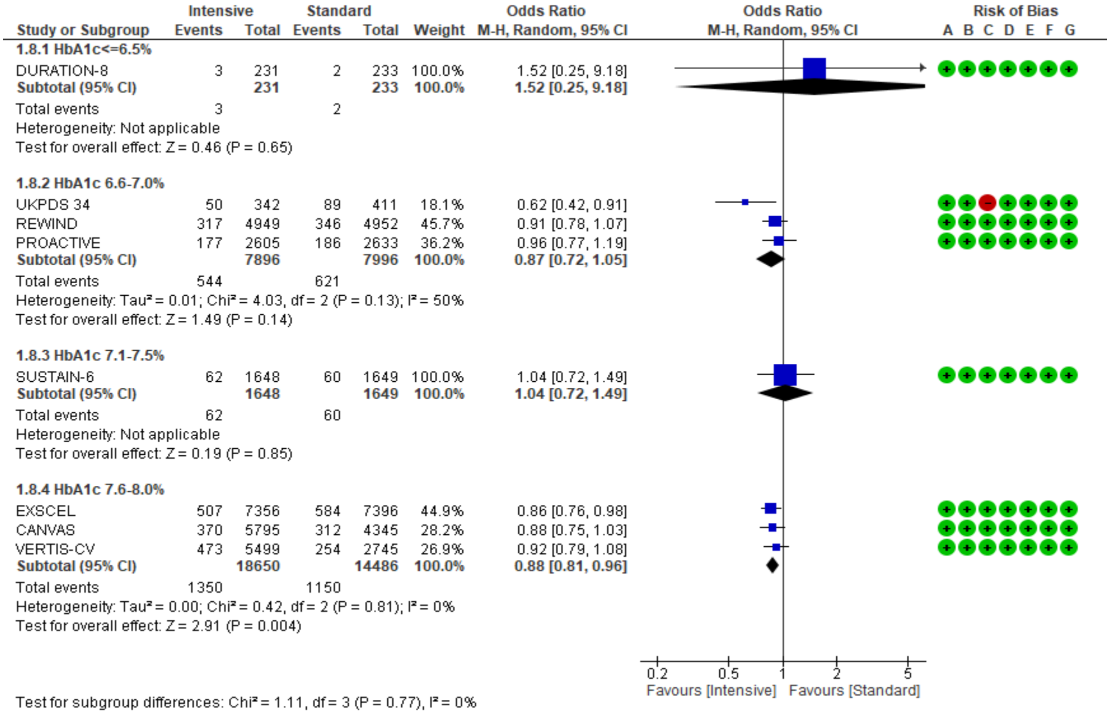

## All-cause mortality

**Figure 3** – Forest plot for trials comparing the effects of intensive glycemic control (using drugs associated with hypoglycemia) and standard care on all-cause mortality.

| Study or Subgroup | Intensive Events | Intensive Total | Standard Events | Standard Total | Weight | Odds Ratio M-H, Random, 95% CI |
|---|---|---|---|---|---|---|
| **1.8.1 HbA1c<=6.5%** | | | | | | |
| **Subtotal (95% CI)** | | **0** | | **0** | | **Not estimable** |
| Total events | 0 | | 0 | | | |
| Heterogeneity: Not applicable | | | | | | |
| Test for overall effect: Not applicable | | | | | | |
| **1.8.2 HbA1c 6.6-7.0%** | | | | | | |
| UKPDS 34 | 50 | 342 | 89 | 411 | 3.1% | 0.62 [0.42, 0.91] |
| REWIND | 317 | 4949 | 346 | 4952 | 18.1% | 0.91 [0.78, 1.07] |
| PROACTIVE | 177 | 2605 | 186 | 2633 | 9.9% | 0.96 [0.77, 1.19] |
| **Subtotal (95% CI)** | | **7896** | | **7996** | **31.1%** | **0.87 [0.72, 1.05]** |
| Total events | 544 | | 621 | | | |
| Heterogeneity: Tau² = 0.01; Chi² = 4.03, df = 2 (P = 0.13); I² = 50% | | | | | | |
| Test for overall effect: Z = 1.49 (P = 0.14) | | | | | | |
| **1.8.3 HbA1c 7.1-7.5%** | | | | | | |
| SUSTAIN-6 | 62 | 1648 | 60 | 1649 | 3.4% | 1.04 [0.72, 1.49] |
| **Subtotal (95% CI)** | | **1648** | | **1649** | **3.4%** | **1.04 [0.72, 1.49]** |
| Total events | 62 | | 60 | | | |
| Heterogeneity: Not applicable | | | | | | |
| Test for overall effect: Z = 0.19 (P = 0.85) | | | | | | |
| **1.8.4 HbA1c 7.6-8.0%** | | | | | | |
| EXSCEL | 507 | 7356 | 584 | 7396 | 29.4% | 0.86 [0.76, 0.98] |
| CANVAS | 370 | 5795 | 312 | 4345 | 18.5% | 0.88 [0.75, 1.03] |
| VERTIS-CV | 473 | 5499 | 254 | 2745 | 17.6% | 0.92 [0.79, 1.08] |
| **Subtotal (95% CI)** | | **18650** | | **14486** | **65.5%** | **0.88 [0.81, 0.96]** |
| Total events | 1350 | | 1150 | | | |
| Heterogeneity: Tau² = 0.00; Chi² = 0.42, df = 2 (P = 0.81); I² = 0% | | | | | | |
| Test for overall effect: Z = 2.91 (P = 0.004) | | | | | | |
| **Total (95% CI)** | | **28194** | | **24131** | **100.0%** | **0.89 [0.83, 0.95]** |
| Total events | 1956 | | 1831 | | | |
| Heterogeneity: Tau² = 0.00; Chi² = 5.14, df = 6 (P = 0.53); I² = 0% | | | | | | |
| Test for overall effect: Z = 3.37 (P = 0.0008) | | | | | | |
| Test for subgroup differences: Chi² = 0.77, df = 2 (P = 0.68), I² = 0% | | | | | | |

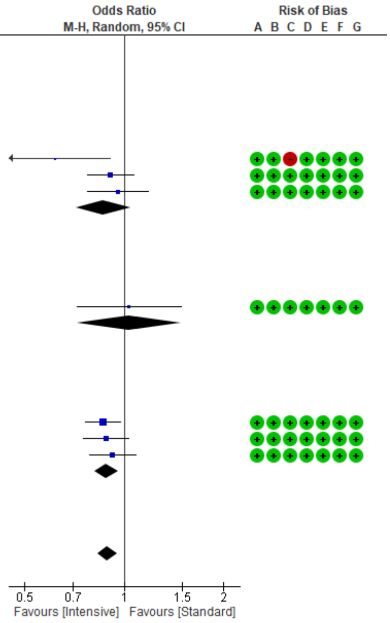


Risk of bias legend
(A) Random sequence generation (selection bias)
(B) Allocation concealment (selection bias)
(C) Blinding of participants and personnel (performance bias)
(D) Blinding of outcome assessment (detection bias)
(E) Incomplete outcome data (attrition bias)
(F) Selective reporting (reporting bias)
(G) Other bias

## GRADE evidence

### Table 1

| Certainty assessment | | | | | | | Summary of findings | | | | |
|---|---|---|---|---|---|---|---|---|---|---|---|
| Participants (studies) Follow up | Risk of bias | Inconsistency | Indirectness | Imprecision | Publication bias | Overall certainty of evidence | Study event rates (%) | | Relative effect (95% CI) | Anticipated absolute effects | |
| | | | | | | | With Standard care | With Intensive glycemic control | | Risk with placebo | Risk difference with Intensive glycemic control |
| **MACE**<br>*For HbA1c ≤48 mmol/mol (6.5%)* | | | | | | | | | | | |
| 464 (1 RCTs) | not serious | not serious | not serious[c] | very serious | none | ⨁⨁◯◯ LOW | 4/231 (1.7%) | 4/233 (1.7%) | OR 1.01 (0.25;4.08) | - | - |
| **All-cause mortality**<br>*For HbA1c ≤48 mmol/mol (6.5%)* | | | | | | | | | | | |
| 464 (1 RCTs) | not serious | not serious | not serious[c] | very serious | none | ⨁⨁◯◯ LOW | 3/231 (1.3%) | 2/233 (0,85%) | OR 1.52 (0.25 to 9.18) | - | - |

## Pharmacoeconomic evaluations

The search for pharmaeconomic studies has been updated without retrieving further studies for any of the questions included. Please see the previous version of the guidelines[1,2].

# RECOMMENDATION # 2: NUTRITIONAL THERAPY.

## Pharmacoeconomic evaluations

The search for pharmaeconomic studies has been updated without retrieving further studies for any of the questions included. Please see the previous version of the guidelines[1,2].

### 2.2 Low carbohydrate vs balanced (Mediterranean) diet

**Figure 4** – Effects of Mediterranean diet in comparison with other standard diets on the risk of incident MACE.

| Study or Subgroup | log[Odds Ratio] | SE | Mediterranean Diet Total | Control Diet Total | Weight | Odds Ratio IV, Random, 95% CI |
|---|---|---|---|---|---|---|
| Delgado-Lista 2022 | -0.2614 | 0.1717 | 256 | 284 | 49.1% | 0.77 [0.55, 1.08] |
| Estruch 2013 | -0.3425 | 0.1688 | 2425 | 1189 | 50.9% | 0.71 [0.51, 0.99] |
| **Total (95% CI)** | | | **2681** | **1473** | **100.0%** | **0.74 [0.58, 0.94]** |

Heterogeneity: Tau² = 0.00; Chi² = 0.11, df = 1 (P = 0.74); I² = 0%
Test for overall effect: Z = 2.51 (P = 0.01)

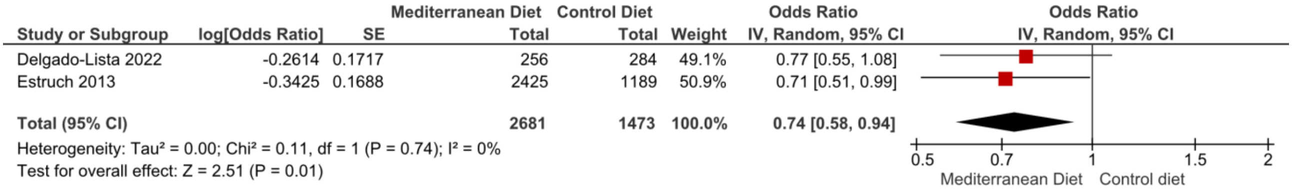

# RECOMMENDATION # 3: PHYSICAL EXERCISE.

## 3.1. Regular physical exercise

## HbA1c

Figure 5 – Forest plot for trials comparing the effects of regular physical exercise and no intervention on HbA1c (%) at endpoint.

| | Aerobic | | | Control | | | | Mean Difference |
|---|---|---|---|---|---|---|---|---|
| Study or Subgroup | Mean | SD | Total | Mean | SD | Total | Weight | IV, Random, 95% CI |
| Andrews 2011 | 6.6 | 0.9 | 246 | 6.7 | 0.9 | 348 | 6.0% | -0.10 [-0.25, 0.05] |
| Balducci 2010 | 6.7 | 1.1 | 303 | 7 | 1.2 | 303 | 5.7% | -0.30 [-0.48, -0.12] |
| Balducci 2019 | 7.1 | 1.6 | 150 | 7.5 | 1.4 | 150 | 4.3% | -0.40 [-0.74, -0.06] |
| Byrkjeland 2015 | 7.2 | 0.8 | 52 | 7.4 | 0.8 | 62 | 4.7% | -0.20 [-0.49, 0.09] |
| Chen 2019 | 7.1 | 0.8 | 36 | 7.2 | 0.8 | 34 | 4.1% | -0.10 [-0.47, 0.27] |
| Cheung 2009 | 7.1 | 1.6 | 20 | 7.7 | 1 | 17 | 1.6% | -0.60 [-1.45, 0.25] |
| Choi 2012 | 6.9 | 0.8 | 38 | 6.9 | 0.7 | 37 | 4.3% | 0.00 [-0.34, 0.34] |
| Church 2010 | 7.3 | 0.7 | 208 | 7.7 | 0.6 | 37 | 5.4% | -0.40 [-0.62, -0.18] |
| Dede 2015 | 6.7 | 1 | 30 | 6.8 | 1 | 30 | 3.1% | -0.10 [-0.61, 0.41] |
| Ghardashi-Afousi A 2020 | 6.8 | 0.8 | 37 | 8 | 1 | 37 | 3.8% | -1.20 [-1.61, -0.79] |
| Gholami 2021 | 8.1 | 1.4 | 14 | 10.1 | 1.5 | 12 | 1.0% | -2.00 [-3.12, -0.88] |
| Gulsin 2020 | 7.3 | 1.1 | 22 | 7.2 | 1.1 | 30 | 2.5% | 0.10 [-0.51, 0.71] |
| Hangping 2019 | 6.7 | 0.9 | 165 | 6.8 | 1.2 | 100 | 4.9% | -0.10 [-0.37, 0.17] |
| Jeon 2020 | 7.2 | 1 | 30 | 7.3 | 1 | 15 | 2.4% | -0.10 [-0.72, 0.52] |
| Kadoglou 2007 | 7.2 | 1 | 30 | 8.1 | 0.9 | 30 | 3.3% | -0.90 [-1.38, -0.42] |
| Kadoglou 2012 | 7.1 | 1 | 27 | 7.9 | 1.1 | 27 | 2.8% | -0.80 [-1.36, -0.24] |
| Kadoglou 2013 | 7.7 | 1.1 | 45 | 7.8 | 0.8 | 24 | 3.4% | -0.10 [-0.55, 0.35] |
| Kwon 2010 | 7 | 0.9 | 13 | 7.3 | 0.9 | 15 | 2.2% | -0.30 [-0.97, 0.37] |
| Magalhães 2019 | 7.2 | 1 | 25 | 7.4 | 1 | 27 | 2.9% | -0.20 [-0.74, 0.34] |
| Mohammadi 2022 | 7.1 | 0.5 | 24 | 7.5 | 0.7 | 12 | 3.5% | -0.40 [-0.84, 0.04] |
| Otten 2017 | 6.2 | 0.8 | 14 | 6.2 | 0.8 | 15 | 2.6% | 0.00 [-0.58, 0.58] |
| Rech 2019 | 6.7 | 0.5 | 17 | 7.3 | 1 | 21 | 3.2% | -0.60 [-1.09, -0.11] |
| Rehman_2017 | 7.3 | 1.4 | 51 | 8.2 | 1.4 | 51 | 2.9% | -0.90 [-1.44, -0.36] |
| Sigal 2007 | 7 | 1.5 | 188 | 7.5 | 1.5 | 63 | 3.6% | -0.50 [-0.93, -0.07] |
| Stubbs 2019 | 7.6 | 1.2 | 33 | 7.7 | 1 | 12 | 2.1% | -0.10 [-0.80, 0.60] |
| Taheri 2020 | 6 | 0.8 | 79 | 6.6 | 0.9 | 79 | 5.0% | -0.60 [-0.87, -0.33] |
| Tessier 2000 | 7.6 | 1.2 | 19 | 7.8 | 1.5 | 20 | 1.6% | -0.20 [-1.05, 0.65] |
| Vidanage 2022 | 7.6 | 1.5 | 75 | 8.3 | 1.1 | 75 | 3.7% | -0.70 [-1.12, -0.28] |
| Vieira 2021 | 7 | 1 | 8 | 7 | 1 | 6 | 1.1% | 0.00 [-1.06, 1.06] |
| Way 2020 | 6.9 | 1 | 11 | 8 | 0.5 | 12 | 2.3% | -1.10 [-1.76, -0.44] |
| **Total (95% CI)** | | | **2010** | | | **1701** | **100.0%** | **-0.39 [-0.51, -0.27]** |

Heterogeneity: Tau² = 0.06; Chi² = 77.57, df = 29 (P < 0.00001); I² = 63%
Test for overall effect: Z = 6.21 (P < 0.00001)

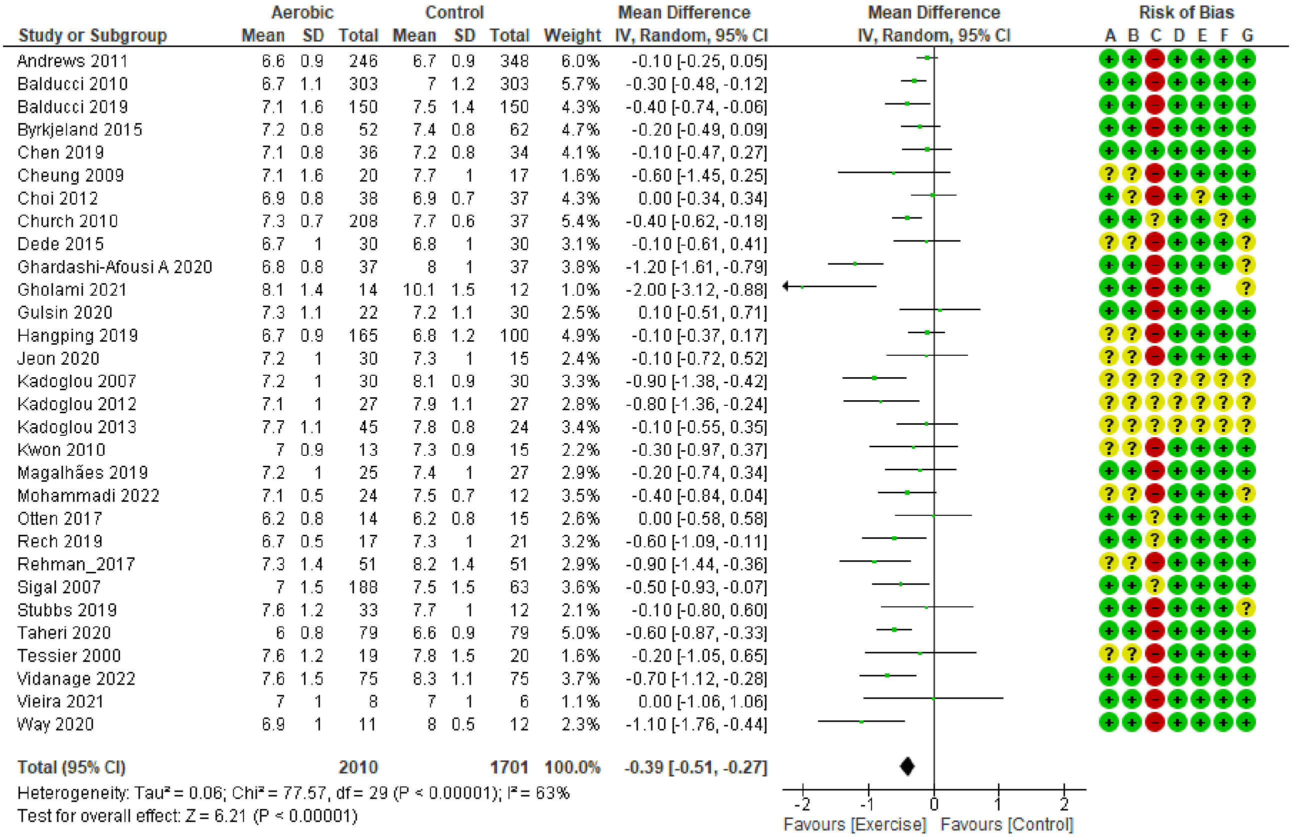


Risk of bias legend
(A) Random sequence generation (selection bias)
(B) Allocation concealment (selection bias)
(C) Blinding of participants and personnel (performance bias)
(D) Blinding of outcome assessment (detection bias)
(E) Incomplete outcome data (attrition bias)
(F) Selective reporting (reporting bias)
(G) Other bias

## Body fat

**Figure 6** – Forest plot for trials comparing the effects of regular physical exercise and no intervention on body fat (%) at endpoint.

| Study or Subgroup | Aerobic Mean | Aerobic SD | Aerobic Total | Control Mean | Control SD | Control Total | Weight | Mean Difference IV, Random, 95% CI |
|---|---|---|---|---|---|---|---|---|
| Andrews 2011 | 33 | 9 | 146 | 35 | 8 | 347 | 21.4% | -2.00 [-3.69, -0.31] |
| Choi 2012 | 32.9 | 6 | 38 | 35.3 | 5 | 37 | 9.8% | -2.40 [-4.90, 0.10] |
| Church 2010 | 36.5 | 7.6 | 69 | 38.6 | 7 | 37 | 7.3% | -2.10 [-4.98, 0.78] |
| Dede 2015 | 31.6 | 8 | 30 | 34.5 | 6.6 | 30 | 4.4% | -2.90 [-6.61, 0.81] |
| de Oliveira 2012 | 34.6 | 15 | 28 | 35.3 | 12.6 | 22 | 1.0% | -0.70 [-8.35, 6.95] |
| Jeon 2020 | 35.8 | 5.9 | 30 | 36.5 | 5.5 | 15 | 5.0% | -0.70 [-4.19, 2.79] |
| Kadoglou 2007 | 35.4 | 7.1 | 30 | 40.7 | 6.9 | 30 | 4.8% | -5.30 [-8.84, -1.76] |
| Kwon 2010 | 32.8 | 9 | 13 | 35.6 | 9 | 15 | 1.4% | -2.80 [-9.48, 3.88] |
| Mohammadi 2022 | 30.2 | 2.1 | 24 | 31.3 | 2.5 | 12 | 22.5% | -1.10 [-2.75, 0.55] |
| Otten 2017 | 33.6 | 6 | 14 | 34.3 | 7 | 15 | 2.7% | -0.70 [-5.44, 4.04] |
| Sigal 2007 | 48 | 5.6 | 60 | 48.3 | 5.6 | 63 | 15.5% | -0.30 [-2.28, 1.68] |
| Taheri 2020 | 28.5 | 12 | 79 | 33.1 | 12.5 | 79 | 4.2% | -4.60 [-8.42, -0.78] |
| Vieira 2021 | 36 | 14 | 34 | 11 | 0 | 0 | | Not estimable |
| **Total (95% CI)** | | | **595** | | | **702** | **100.0%** | **-1.79 [-2.57, -1.01]** |

Heterogeneity: Tau² = 0.00; Chi² = 10.12, df = 11 (P = 0.52); I² = 0%
Test for overall effect: Z = 4.49 (P < 0.00001)

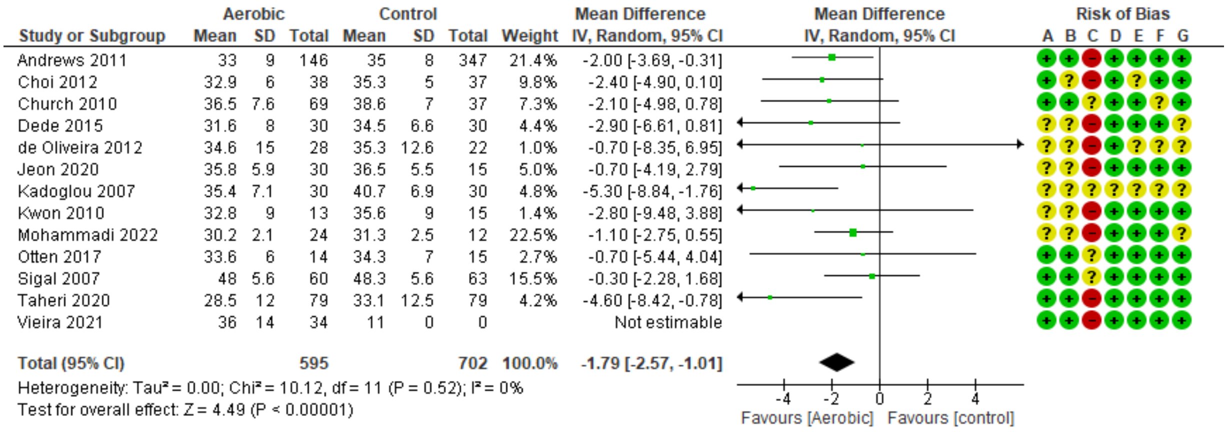


Risk of bias legend
(A) Random sequence generation (selection bias)
(B) Allocation concealment (selection bias)
(C) Blinding of participants and personnel (performance bias)
(D) Blinding of outcome assessment (detection bias)
(E) Incomplete outcome data (attrition bias)
(F) Selective reporting (reporting bias)
(G) Other bias

## BMI

**Figure 7** – Forest plot for trials comparing the effects of regular physical exercise and no intervention on BMI (Kg/m$^2$) at endpoint.

| Study or Subgroup | Aerobic Mean | Aerobic SD | Aerobic Total | Control Mean | Control SD | Control Total | Weight | Mean Difference IV, Random, 95% CI |
|---|---|---|---|---|---|---|---|---|
| Madden 2009 | 29.9 | 4.2 | 17 | 27.7 | 4.2 | 12 | 1.3% | 2.20 [-0.90, 5.30] |
| Vieira 2021 | 31 | 6 | 8 | 29 | 7 | 6 | 0.3% | 2.00 [-4.98, 8.98] |
| Kadoglou 2007 | 32 | 3 | 30 | 30.7 | 3.6 | 30 | 4.1% | 1.30 [-0.38, 2.98] |
| Cheung 2009 | 39.5 | 9 | 20 | 38.2 | 9.2 | 17 | 0.4% | 1.30 [-4.59, 7.19] |
| Tessier 2000 | 30.6 | 5.4 | 19 | 29.4 | 3.8 | 20 | 1.4% | 1.20 [-1.74, 4.14] |
| Jennings 2009 | 31.8 | 5.3 | 50 | 31.5 | 4.8 | 22 | 2.0% | 0.30 [-2.19, 2.79] |
| Otten 2017 | 29.6 | 4.5 | 14 | 29.4 | 4.5 | 15 | 1.2% | 0.20 [-3.08, 3.48] |
| de Oliveira 2012 | 30.1 | 3 | 31 | 30 | 4.9 | 12 | 1.4% | 0.10 [-2.87, 3.07] |
| Stubbs 2019 | 31.9 | 4.4 | 33 | 31.8 | 4.1 | 12 | 1.6% | 0.10 [-2.66, 2.86] |
| Sigal 2007 | 34.2 | 4.8 | 188 | 34.2 | 4.8 | 63 | 5.8% | 0.00 [-1.37, 1.37] |
| Kadoglou 2013 | 32.2 | 3.1 | 66 | 32.3 | 2.9 | 24 | 5.7% | -0.10 [-1.48, 1.28] |
| Way 2020 | 36 | 4.5 | 11 | 36.2 | 6.2 | 12 | 0.7% | -0.20 [-4.60, 4.20] |
| Mohammadi 2022 | 27.8 | 2.2 | 12 | 28.2 | 2.5 | 12 | 3.3% | -0.40 [-2.28, 1.48] |
| Magalhães 2019 | 30.1 | 5.4 | 53 | 30.7 | 4.9 | 27 | 2.2% | -0.60 [-2.95, 1.75] |
| Kwon 2010 | 26.7 | 2.2 | 15 | 27.3 | 2.8 | 12 | 3.2% | -0.60 [-2.54, 1.34] |
| Dede 2015 | 30 | 4.5 | 30 | 30.9 | 4.7 | 30 | 2.3% | -0.90 [-3.23, 1.43] |
| Jeon 2020 | 24.4 | 2.7 | 30 | 25.3 | 2.4 | 15 | 4.7% | -0.90 [-2.45, 0.65] |
| Kadoglou 2012 | 31.3 | 3.1 | 27 | 32.3 | 2.9 | 27 | 4.5% | -1.00 [-2.60, 0.60] |
| Ghardashi-Afousi A 2020 | 28.2 | 1.1 | 37 | 29.4 | 1.2 | 37 | 20.3% | -1.20 [-1.72, -0.68] |
| Kwon 2010a | 26.2 | 2.3 | 14 | 27.4 | 2.9 | 12 | 2.9% | -1.20 [-3.24, 0.84] |
| Church 2010 | 34 | 5.9 | 208 | 35.2 | 6.2 | 37 | 2.6% | -1.20 [-3.35, 0.95] |
| Balducci 2019 | 28.8 | 4.9 | 150 | 30.1 | 5.3 | 150 | 7.7% | -1.30 [-2.46, -0.14] |
| Balducci 2010 | 30.3 | 4.4 | 303 | 31.7 | 4.5 | 303 | 15.0% | -1.40 [-2.11, -0.69] |
| Gulsin 2020 | 33 | 5.5 | 22 | 34.5 | 5.5 | 35 | 1.5% | -1.50 [-4.43, 1.43] |
| Taheri 2020 | 31 | 5.2 | 79 | 33.7 | 5.8 | 79 | 3.9% | -2.70 [-4.42, -0.98] |
| **Total (95% CI)** | | | **1467** | | | **1021** | **100.0%** | **-0.79 [-1.15, -0.42]** |

Heterogeneity: Tau² = 0.10; Chi² = 27.52, df = 24 (P = 0.28); I² = 13%
Test for overall effect: Z = 4.26 (P < 0.0001)

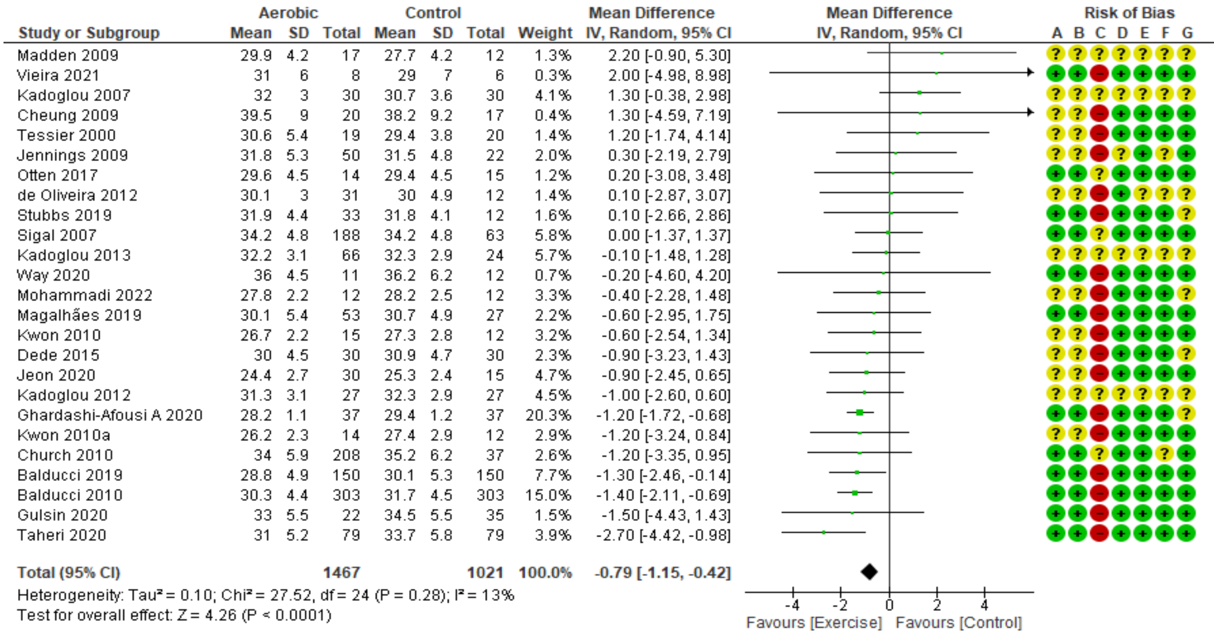


Risk of bias legend
(A) Random sequence generation (selection bias)
(B) Allocation concealment (selection bias)
(C) Blinding of participants and personnel (performance bias)
(D) Blinding of outcome assessment (detection bias)
(E) Incomplete outcome data (attrition bias)
(F) Selective reporting (reporting bias)
(G) Other bias

## GRADE evidence table

### Table 2

| Certainty assessment | | | | | | | Summary of findings | | |
|---|---|---|---|---|---|---|---|---|---|
| Participants (studies) Follow up | Risk of bias | Inconsistency | Indirectness | Imprecision | Publication bias | Overall certainty of evidence | Relative effect (95%, CI) | Anticipated absolute effects: Control | Anticipated absolute effects: Intervention |
| **HbA1c (%)** | | | | | | | | | |
| 3711 (30 RCT) | serious[a] | not serious | not serious | not serious | Strong association | ⨁⨁⨁⨁ HIGH | **-0.39 [-0.51, -.27]** | Mean HbA1c at endpoint: 7.4 % | **MD 0.39 % lower** (from 0.51 lower to 0.27 lower) |
| **Body fat percentage at endpoint (%)** | | | | | | | | | |
| 1297 (13 RCT) | serious[a] | not serious | not serious | not serious | Strong association; possible publication bias | ⨁⨁⨁◯ MODERATE | **-1.79 [-2.57, -1.01]** | Mean body fat at endpoint:: 34.7% | **MD 1.79 % lower** (2.57 lower to 1.01 lower) |
| **BMI** (Kg/m$^2$) | | | | | | | | | |
| 2488 (25 RCT) | serious[a] | not serious | not serious | serious[b] | Strong association; possible publication bias | ⨁⨁⨁⨁ HIGH | **-0.79 [-1.15,-0.42]** | Mean BMI at endpoint: 31.0 Kg/m$^2$ | **MD 0.8 Kg/m$^2$ lower** (da 1.1 a 0.4 lower) |

**CI:** Confidence interval; **MD:** Mean difference; a. Randomization, allocation, and blinding procedures not adequately reported for the majority of included trials; b. Limited sample size.

## 3.2. Duration of aerobic exercise

## HbA1c

**Figure 8** – Forest plot for trials comparing the effects of regular aerobic physical exercise (versus no intervention), with a duration> or ≤150 min/week on HbA1c (%) at endpoint.

| Study or Subgroup | Aerobic Mean | Aerobic SD | Aerobic Total | Control Mean | Control SD | Control Total | Weight | Mean Difference IV, Random, 95% CI |
|---|---|---|---|---|---|---|---|---|
| **5.7.1 >=150 min** | | | | | | | | |
| Kadoglou 2007 | 7.2 | 1 | 30 | 8.1 | 0.9 | 30 | 3.7% | -0.90 [-1.38, -0.42] |
| Vidanage 2022 | 7.6 | 1.5 | 75 | 8.3 | 1.1 | 75 | 4.4% | -0.70 [-1.12, -0.28] |
| Taheri 2020 | 6 | 0.8 | 79 | 6.6 | 0.9 | 79 | 7.1% | -0.60 [-0.87, -0.33] |
| Church 2010 | 7.3 | 0.7 | 208 | 7.7 | 0.6 | 37 | 8.3% | -0.40 [-0.62, -0.18] |
| Balducci 2010 | 6.7 | 1.1 | 303 | 7 | 1.2 | 303 | 9.1% | -0.30 [-0.48, -0.12] |
| Byrkjeland 2015 | 7.2 | 0.8 | 52 | 7.4 | 0.8 | 62 | 6.5% | -0.20 [-0.49, 0.09] |
| Tessier 2000 | 7.6 | 1.2 | 19 | 7.8 | 1.5 | 20 | 1.5% | -0.20 [-1.05, 0.65] |
| Andrews 2011 | 6.6 | 0.9 | 246 | 6.7 | 0.9 | 348 | 10.0% | -0.10 [-0.25, 0.05] |
| Kadoglou 2013 | 7.7 | 1.1 | 45 | 7.8 | 0.8 | 24 | 4.0% | -0.10 [-0.55, 0.35] |
| Otten 2017 | 6.2 | 0.8 | 14 | 6.2 | 0.8 | 15 | 2.8% | 0.00 [-0.58, 0.58] |
| Choi 2012 | 6.9 | 0.8 | 38 | 6.9 | 0.7 | 37 | 5.7% | 0.00 [-0.34, 0.34] |
| Gulsin 2020 | 7.3 | 1.1 | 22 | 7.2 | 1.1 | 30 | 2.6% | 0.10 [-0.51, 0.71] |
| **Subtotal (95% CI)** | | | **1131** | | | **1060** | **65.7%** | **-0.30 [-0.45, -0.15]** |
| Heterogeneity: Tau² = 0.04; Chi² = 28.63, df = 11 (P = 0.003); I² = 62% | | | | | | | | |
| Test for overall effect: Z = 3.90 (P < 0.0001) | | | | | | | | |
| **5.7.2 <150 min** | | | | | | | | |
| Rehman_2017 | 7.3 | 1.4 | 51 | 8.2 | 1.4 | 51 | 3.1% | -0.90 [-1.44, -0.36] |
| Sigal 2007 | 7 | 1.5 | 188 | 7.5 | 1.5 | 63 | 4.3% | -0.50 [-0.93, -0.07] |
| Balducci 2019 | 7.1 | 1.6 | 150 | 7.5 | 1.4 | 150 | 5.7% | -0.40 [-0.74, -0.06] |
| Mohammadi 2022 | 7.1 | 0.5 | 24 | 7.5 | 0.7 | 12 | 4.1% | -0.40 [-0.84, 0.04] |
| Magalhães 2019 | 7.2 | 1 | 25 | 7.4 | 1 | 27 | 3.1% | -0.20 [-0.74, 0.34] |
| Dede 2015 | 6.7 | 1 | 30 | 6.8 | 1 | 30 | 3.4% | -0.10 [-0.61, 0.41] |
| Hangping 2019 | 6.7 | 0.9 | 165 | 6.8 | 1.2 | 100 | 7.0% | -0.10 [-0.37, 0.17] |
| Jeon 2020 | 7.2 | 1 | 30 | 7.3 | 1 | 15 | 2.5% | -0.10 [-0.72, 0.52] |
| Vieira 2021 | 7 | 1 | 8 | 7 | 1 | 6 | 1.0% | 0.00 [-1.06, 1.06] |
| **Subtotal (95% CI)** | | | **671** | | | **454** | **34.3%** | **-0.31 [-0.47, -0.14]** |
| Heterogeneity: Tau² = 0.01; Chi² = 9.56, df = 8 (P = 0.30); I² = 16% | | | | | | | | |
| Test for overall effect: Z = 3.65 (P = 0.0003) | | | | | | | | |
| **Total (95% CI)** | | | **1802** | | | **1514** | **100.0%** | **-0.30 [-0.41, -0.19]** |
| Heterogeneity: Tau² = 0.03; Chi² = 38.30, df = 20 (P = 0.008); I² = 48% | | | | | | | | |
| Test for overall effect: Z = 5.34 (P < 0.00001) | | | | | | | | |
| Test for subgroup differences: Chi² = 0.01, df = 1 (P = 0.92), I² = 0% | | | | | | | | |

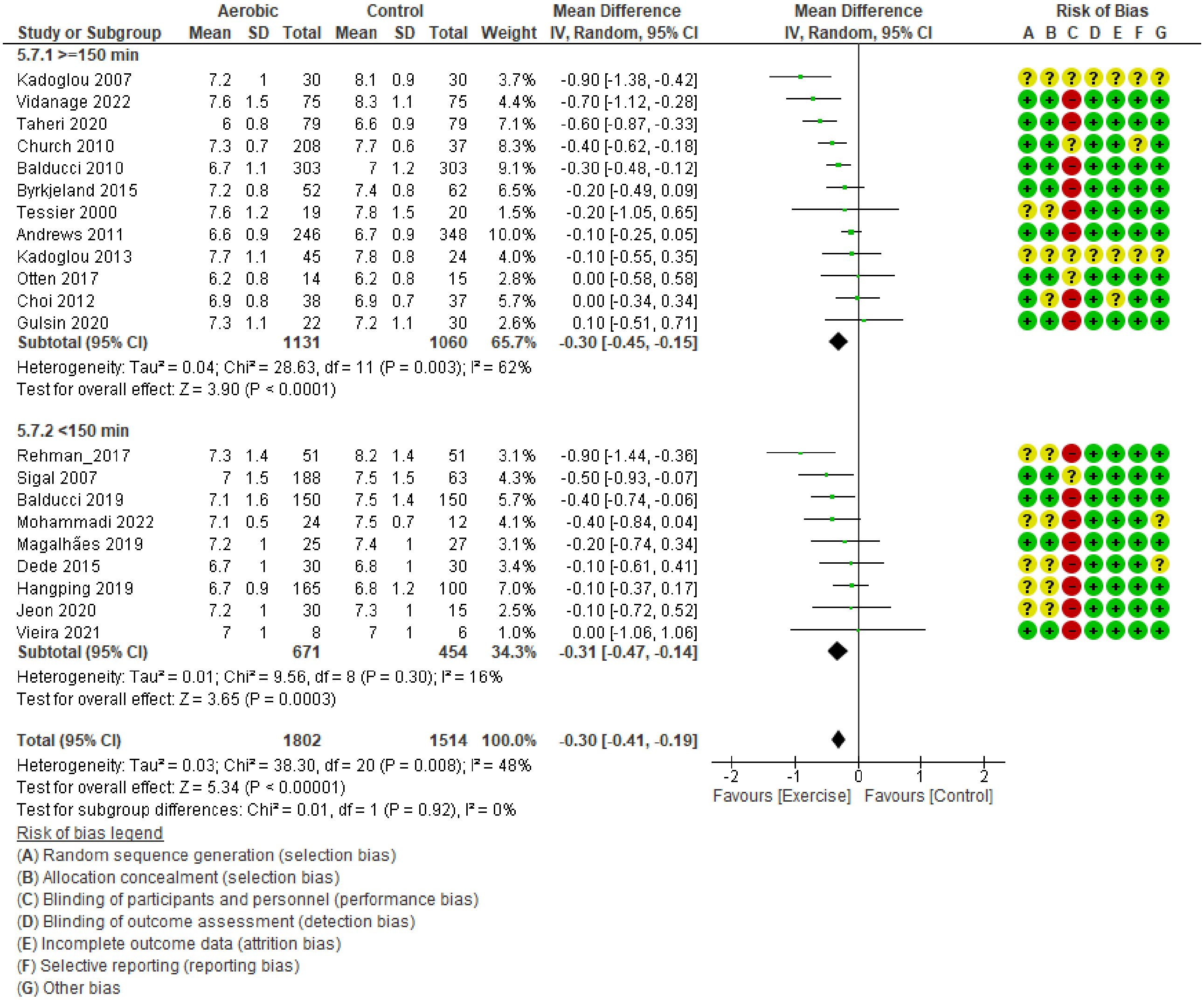


Risk of bias legend
(A) Random sequence generation (selection bias)
(B) Allocation concealment (selection bias)
(C) Blinding of participants and personnel (performance bias)
(D) Blinding of outcome assessment (detection bias)
(E) Incomplete outcome data (attrition bias)
(F) Selective reporting (reporting bias)
(G) Other bias

# Body fat

**Figure 9** – Forest plot for trials comparing the effects of regular aerobic physical exercise (versus no intervention), with a duration> or ≤150 min/week on body fat (%) at endpoint.

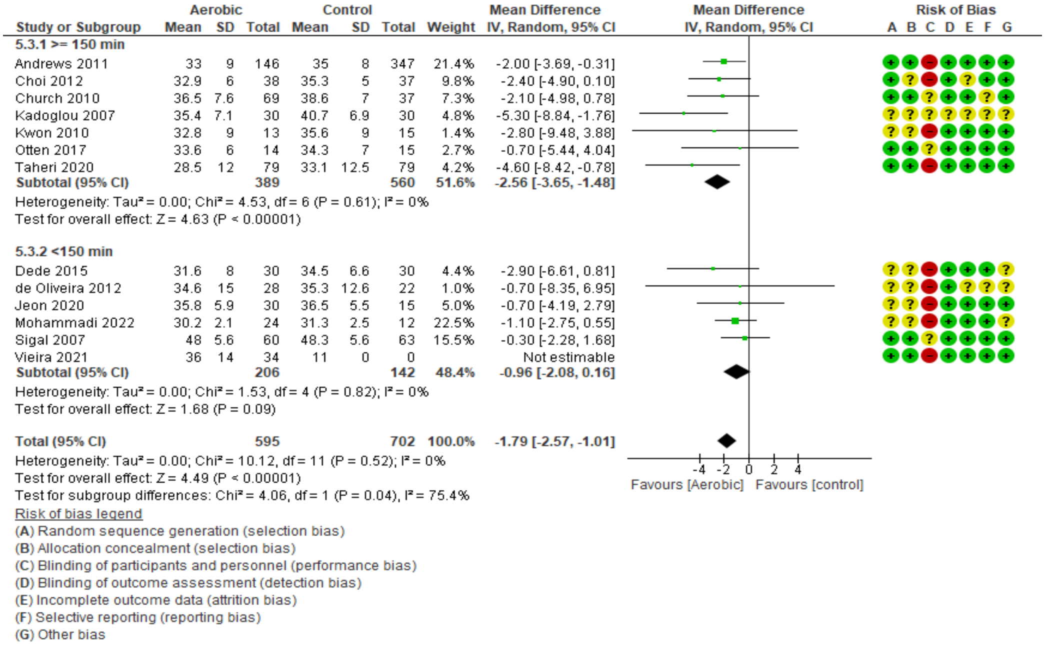


# BMI

**Figure 10** –Forest plot for trials comparing the effects of regular aerobic physical exercise (versus no intervention), with a duration> or ≤150 min/week on BMI (Kg/m$^2$) at endpoint.

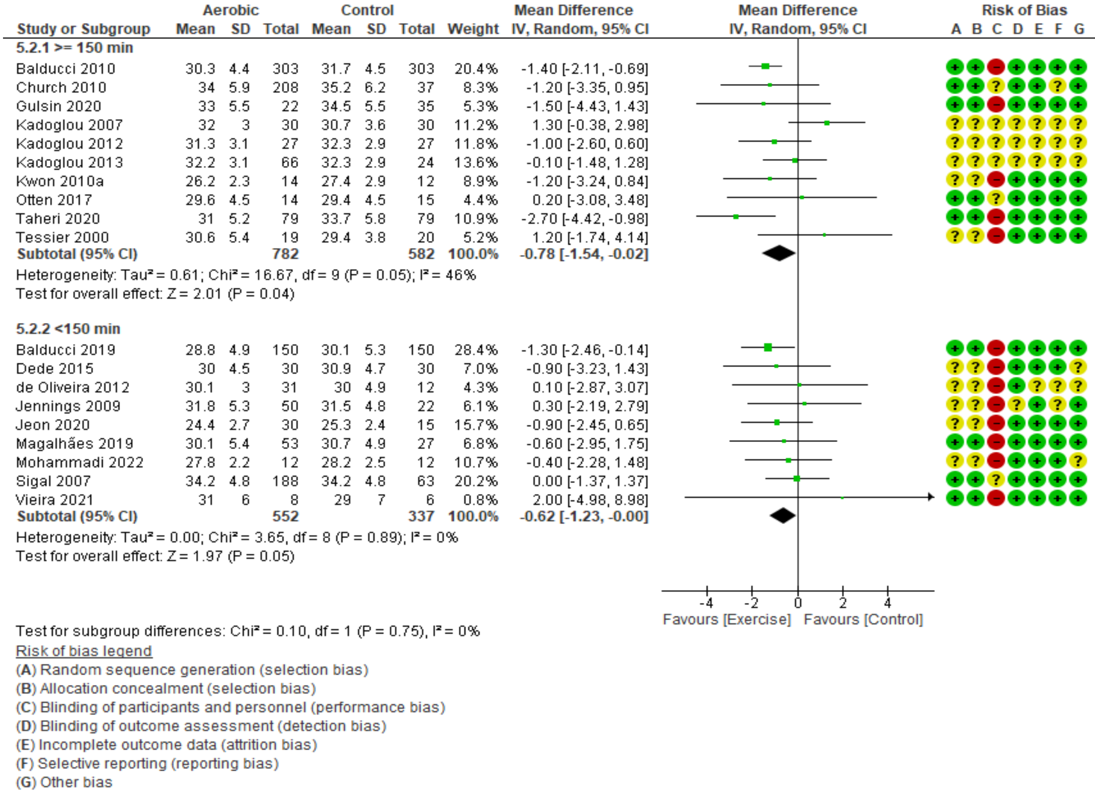

## GRADE evidence

### Table 3

| Certainty assessment | | | | | | | Summary of findings | | |
|---|---|---|---|---|---|---|---|---|---|
| **Participants (studies) Follow up** | **Risk of bias** | **Inconsistency** | **Indirectness** | **Imprecision** | **Publication bias** | **Overall certainty of evidence** | **Relative effect** (95%, CI) | **Anticipated absolute effects** | |
| | | | | | | | | **Control** | **Intervention** |
| **HbA1c** (%) for RCT with physical exercise ≤ 150 min/week | | | | | | | | | |
| 1125 (9 RCT) | serious[a] | not serious | not serious | serious[b] | publication bias[c] | ⊕◯◯◯ VERY LOW | **-0.30** [-0.47;-0.14] | - | MD **0.31 lower** (from 0.47 lower to 0.14 lower) |
| **HbA1c** (Kg/m$^2$) for RCT with physical exercise >150 min/week | | | | | | | | | |
| 2191 (12 RCT) | serious[a] | not serious | not serious | serious[b] | publication bias[c] | ⊕◯◯◯ VERY LOW | **-0.30** [-0.45;-0.15] | - | MD **0.3 lower** (from 0.45 lower to 0.15 lower) |
| **Massa grassa** (%) for RCT with physical exercise ≤ 150 min/week | | | | | | | | | |
| 348 (6 RCT) | serious[a] | not serious | not serious | serious[b] | publication bias[c] | ⊕◯◯◯ VERY LOW | -1.20 [-2.70;0.29] | - | MD 1.2 % lower (from 2.7 lower to 0.3 more) |
| **Massa grassa** (%) per gli studi dii durata >150 min/ settimana | | | | | | | | | |
| 921 (6 RCT) | serious[a] | not serious | not serious | serious[b] | publication bias[c] | ⊕◯◯◯ VERY LOW | **-2.56** [-3.65; -1.48] | - | **MD 2.56 lower (3.66 lower 7o 1.46 lower)** |

**Indice di massa corporea** (Kg/$m^2$) per gli studi dii durata ≤150 min/ settimana

| | | | | | | | | | |
|---|---|---|---|---|---|---|---|---|---|
| 1364 (10 RCT) | serious[a] | not serious | not serious | serious[b] | publication bias[c] | ⨁◯◯◯ VERY LOW | -0.78 [-1.54;-0.02] | - | **MD 0.78 lower (1.54 lower to 0.02 lower)** |

**Indice di massa corporea** (Kg/$m^2$) per gli studi dii durata >150 min/settimana

| | | | | | | | | | |
|---|---|---|---|---|---|---|---|---|---|
| 889 (9 RCT) | serious[a] | not serious | not serious | serious[b] | publication bias[c] | ⨁◯◯◯ VERY LOW | -0.62 [-1.23;0.0] | - | MD **0.62 lower** (1.23 **lower** to 0) |

**CI:** Confidence interval; **MD:** Mean difference; a. Randomization, allocation, and blinding procedures not adequately reported for the majority of included trials; b. Limited sample size; c. Funnel plot showing possible publication bias, confirmed by Egger's test.

## 3.3 Different modalities of physical exercise

## HbA1c

**Figure 11** – Forest plot for trials comparing the effects of combined exercise (aerobic and resistance) and aerobic exercise on HbA1c (%) at endpoint.

| | Combined | | | Aerobic | | | | Mean Difference | Risk of Bias | | | | | | |
|---|---|---|---|---|---|---|---|---|---|---|---|---|---|---|---|
| Study or Subgroup | Mean | SD | Total | Mean | SD | Total | Weight | IV, Random, 95% CI | A | B | C | D | E | F | G |
| Bouchi 2021 | 7.2 | 0.7 | 72 | 7.2 | 0.6 | 69 | 44.9% | 0.00 [-0.21, 0.21] | + | + | - | + | + | + | + |
| Church 2010 | 7.3 | 0.7 | 71 | 7.4 | 0.7 | 69 | 39.1% | -0.10 [-0.33, 0.13] | + | + | ? | + | + | ? | + |
| Kadoglou 2013 | 7.3 | 1 | 22 | 7.7 | 1.1 | 21 | 5.8% | -0.40 [-1.03, 0.23] | ? | ? | ? | ? | ? | ? | ? |
| Sigal 2007 | 6.6 | 1.5 | 64 | 7 | 1.5 | 60 | 8.1% | -0.40 [-0.93, 0.13] | + | + | ? | + | + | + | + |
| Stubbs 2019 | 7.3 | 1 | 11 | 8 | 1.5 | 11 | 2.0% | -0.70 [-1.77, 0.37] | + | + | - | + | + | + | ? |
| **Total (95% CI)** | | | **240** | | | **230** | **100.0%** | **-0.11 [-0.26, 0.04]** | | | | | | | |

Heterogeneity: Tau² = 0.00; Chi² = 4.16, df = 4 (P = 0.38); I² = 4%
Test for overall effect: Z = 1.40 (P = 0.16)

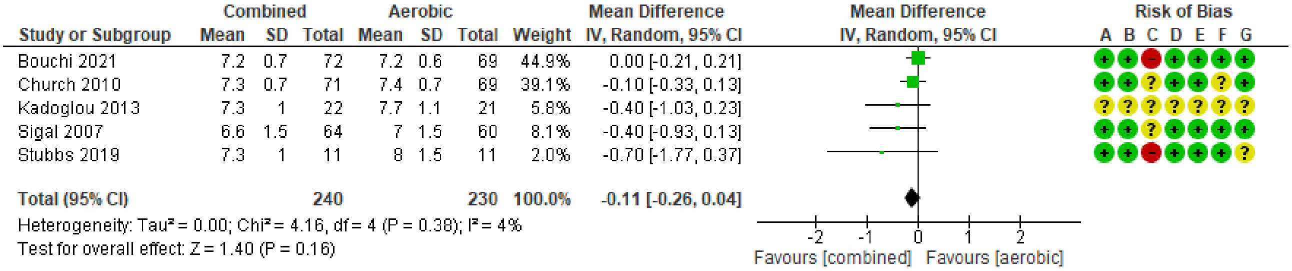


Risk of bias legend
(A) Random sequence generation (selection bias)
(B) Allocation concealment (selection bias)
(C) Blinding of participants and personnel (performance bias)
(D) Blinding of outcome assessment (detection bias)
(E) Incomplete outcome data (attrition bias)
(F) Selective reporting (reporting bias)
(G) Other bias

## GRADE evidence table

### Table 4

| Certainty assessment | | | | | | | Summary of findings | | |
|---|---|---|---|---|---|---|---|---|---|
| **Participants (studies) Follow up** | **Risk of bias** | **Inconsistency** | **Indirectness** | **Imprecision** | **Publication bias** | **Overall certainty of evidence** | **Relative effect** (95%, CI) | **Anticipated absolute effects** | |
| | | | | | | | | **Control** | **Intervention** |
| **HbA1c** (%) | | | | | | | | | |
| 470 (5 RCTs) | serious[a] | not serious | not serious | serious[b] | Possible publication bias[c] | ⨁◯◯◯ VERY LOW | **-0.11** [-0.26, 0.04] | Mean HbA1c at the end of the study: 7.2% | **MD 0.11 % lower** (from 0.26 lower to 0.4 higher) |

**CI:** Confidence interval; **MD:** Mean difference; a. Randomization, allocation, and blinding procedures not adequately reported for the majority of included trials; b. Limited sample size; c. Funnel plot showing possible publication bias, confirmed by Egger's test.

## Pharmacoeconomic evaluations

The search for pharmaeconomic studies has been updated without retrieving further studies for any of the questions included. Please see the previous version of the guidelines[1,2].

## RECOMMENDATION # 5: PHARMACOLOGICAL THERAPY.

**Table 5** – New trials included after updating the previous version of guidelines (20/05/22) for glucometabolic control.

| **First name** (Pub. year) | **Drug 1** | **Drug 2** | **Trial duration** (weeks) | **N. patients** Drug 1 | **N. patients** Drug 2 |
|---|---|---|---|---|---|
| Buse 2020[3] | Sitagliptin | Oral semaglutide | 52 | 98 | 100 |
| Weinstock 2015[4] | Sitagliptin | Dulaglutide | 104 | 315 | 304 |
| Pfutzner 2011[5] | Saxagliptin | Metformin | 76 | 335 | 328 |
| Khaloo 2019[6] | Sitagliptin | Pioglitazone | 52 | 125 | 125 |
| Handelsman 2017[7] | Omarigliptin | Glimepiride | 54 | 376 | 375 |
| Terauchi[8] 2017 | Sitagliptin | Glimepiride | 52 | 143 | 127 |
| Jabbour 2020[9] | Dapagliflozin | Exenatide | 104 | 230 | 227 |
| Lingvay 2019[10] | Canagliflozin | Semaglutide | 52 | 394 | 394 |
| Rodbard 2019[11] | Empagliflozin | Semaglutide | 52 | 410 | 411 |
| Ridderstrale 2018[12] | Empagliflozin | Glimepiride | 208 | 765 | 780 |
| Kaku 2019[13] | Liraglutide | Degludec | 52 | 273 | 271 |
| Wang 2019[14] | Dulaglutide | Glargine | 52 | 253 | 250 |
| Zhang 2020[15] | Exenatide | Insulin | 52 | 27 | 32 |

## HbA1c

**Figure 12** – RCTs comparing different glucose-lowering agents versus other active drugs, with a duration ≥ of 52 weeks[16].

Network metanalysis of different glucose-lowering agents: forest plots of comparisons versus metformin. Panel A: 52 weeks; Panel B: ≥104 weeks. GLP-1 RA: Glucagon-Like Peptide-1 Receptor Agonists; SGLT-2: Sodium-Glucose Transporter-2; Sulfonylureas include also glinides.

**A**

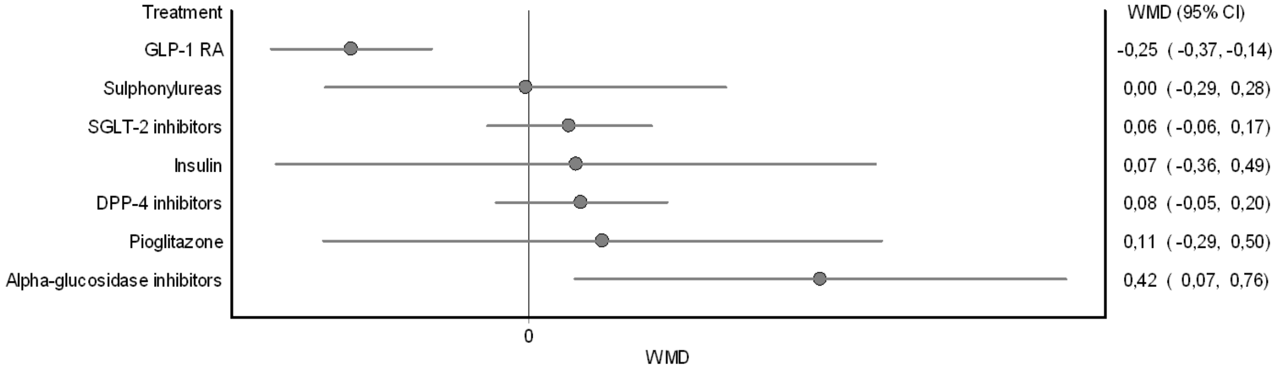


*Consistency H: 1.55*

**B**

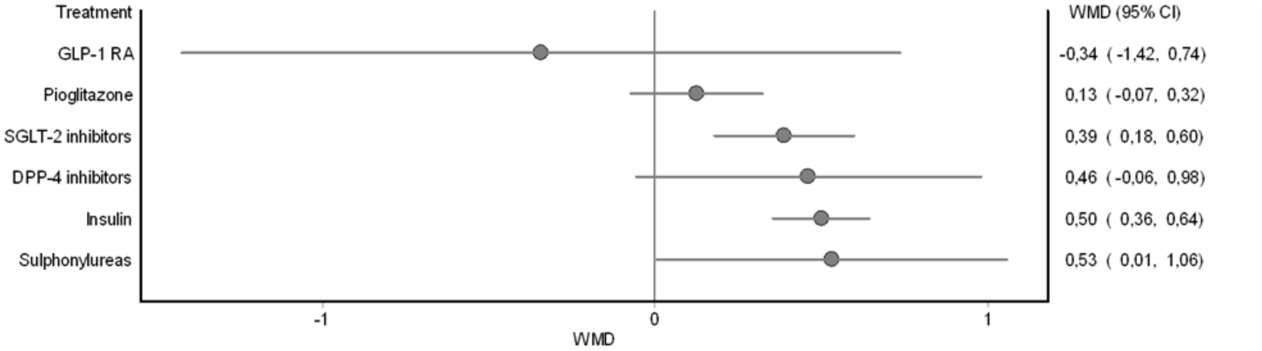


*Consistency H:1.68*

## BMI

**Figure 13** – Network metanalysis of different glucose-lowering agents: forest plots of comparisons versus metformin for BMI at endpoint [16].

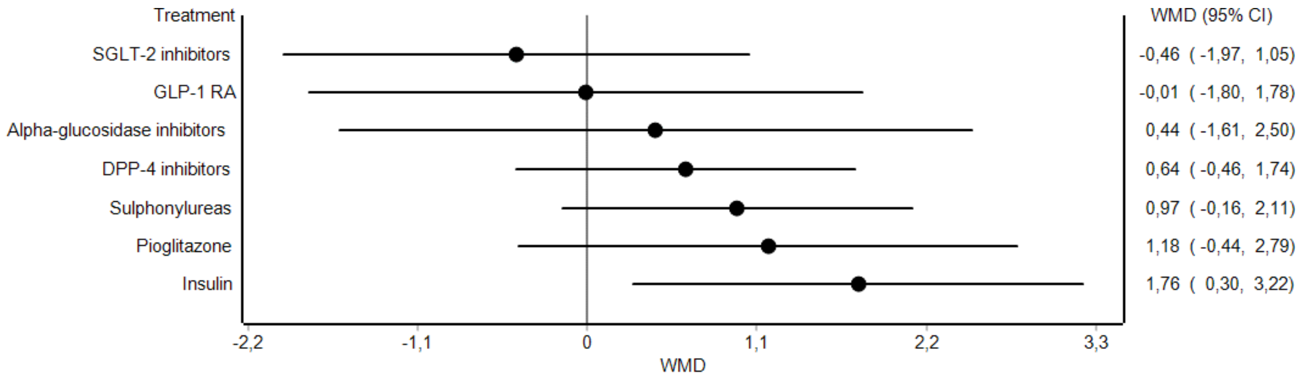


*Consistency H: 2.056*

## Severe hypoglycemia

**Figure 14** – Network metanalysis of different glucose-lowering agents: forest plots of comparisons versus metformin for severe hypoglycemia[16].

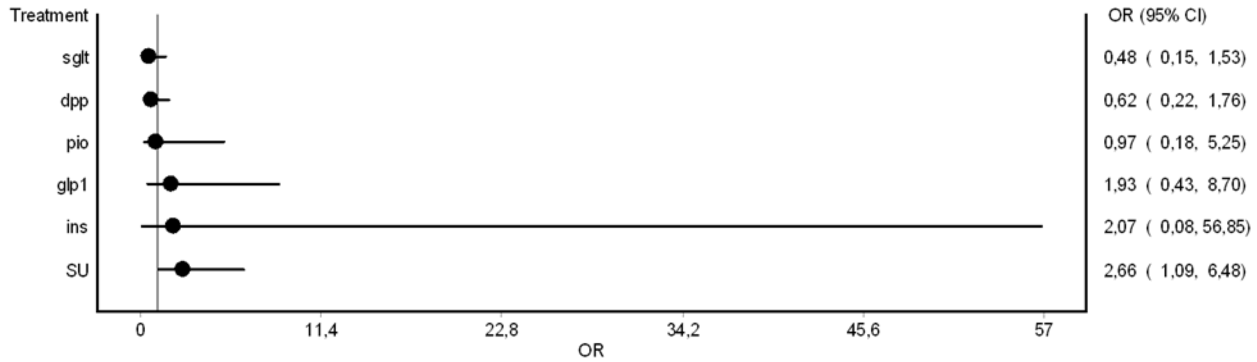


*Consistency H: 1.001*

## 5.4. Pharmacoeconomic evaluations

The search for pharmaeconomic studies has been updated without retrieving further studies for any of the questions included. Please see the previous version of the guidelines[1,2].

## 5.6. Long-acting basal analogues

**EVIDENCES**

This recommendation is based on results of an unpublished meta-analysis updated up to 01/05/2022.

*Pubmed*
*("insulin detemir"[MeSH Terms] OR ("insulin"[All Fields] AND "detemir"[All Fields]) OR "insulin detemir"[All Fields] OR "detemir"[All Fields]) OR ("insulin glargine"[MeSH Terms] OR ("insulin"[All Fields] AND "glargine"[All Fields]) OR "insulin glargine"[All Fields] OR "glargine"[All Fields]) OR ("insulin degludec"[Supplementary Concept] OR "insulin degludec"[All Fields] OR "degludec"[All Fields]) OR aspart[All Fields] OR ("insulin lispro"[MeSH Terms] OR ("insulin"[All Fields] AND "lispro"[All Fields]) OR "insulin lispro"[All Fields] OR "lispro"[All Fields]) OR glulisine[All Fields] AND Randomized Controlled Trial[ptyp] AND (Randomized Controlled Trial[ptyp] AND "humans"[MeSH Terms])*
*Embase*
*'detemir'/exp OR detemir OR 'glargine'/exp OR glargine OR 'degludec'/exp OR degludec OR 'aspart'/exp OR aspart OR 'lispro'/exp OR lispro OR 'glulisine'/exp OR glulisine AND [embase]/lim NOT ([embase]/lim AND [medline]/lim) AND 'randomized controlled trial'/de AND 'humans'/de.*
*Cochrane Library*
*Trials matching detemir or glargine or degludec or aspart or lispro or glulisine in Title Abstract Keyword - in Trials (Word variations have been searched) [Source: The International Clinical Trial Registry Platform].*

**Figure 15 –** Forest plot for trials comparing the effects of long-acting basal insulin with longer vs. shorter duration on total hypoglycemic risk.

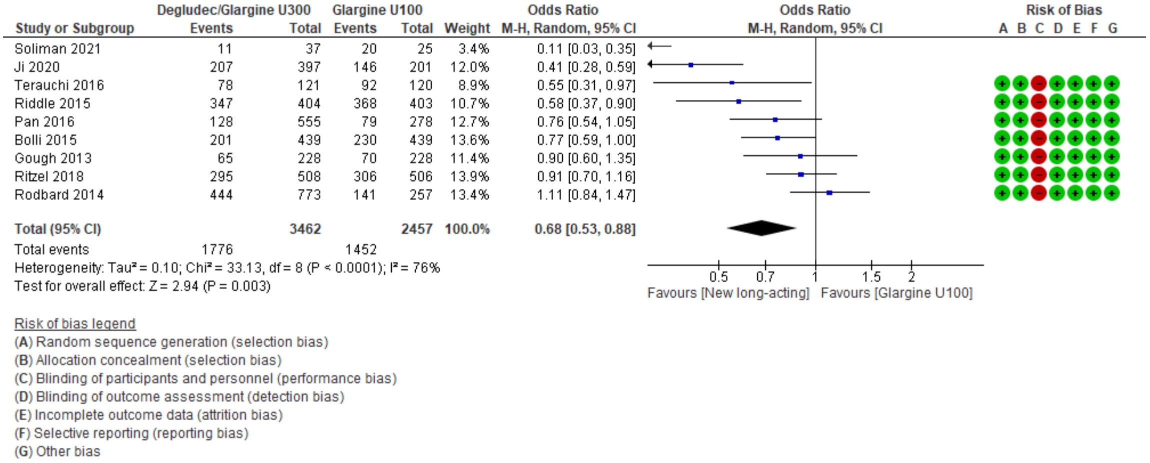


**Figure 16 –** Forest plot for trials comparing the effects of long-acting basal insulin with longer vs. shorter duration on nocturnal hypoglycemic risk.

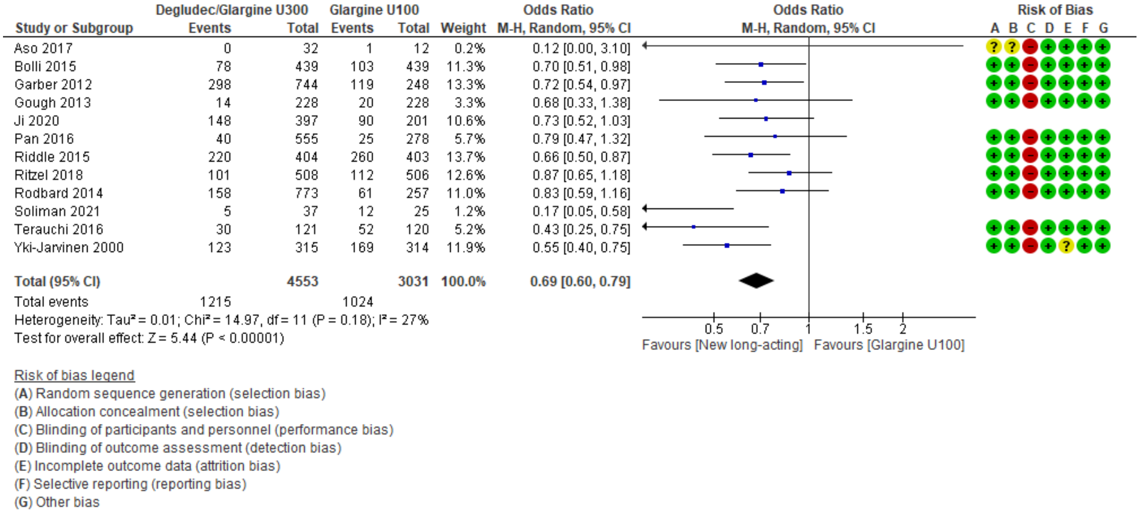


**Figure 17 –** Forest plot for trials comparing the effects of long-acting basal insulin with longer vs. shorter duration on severe hypoglycemic risk.

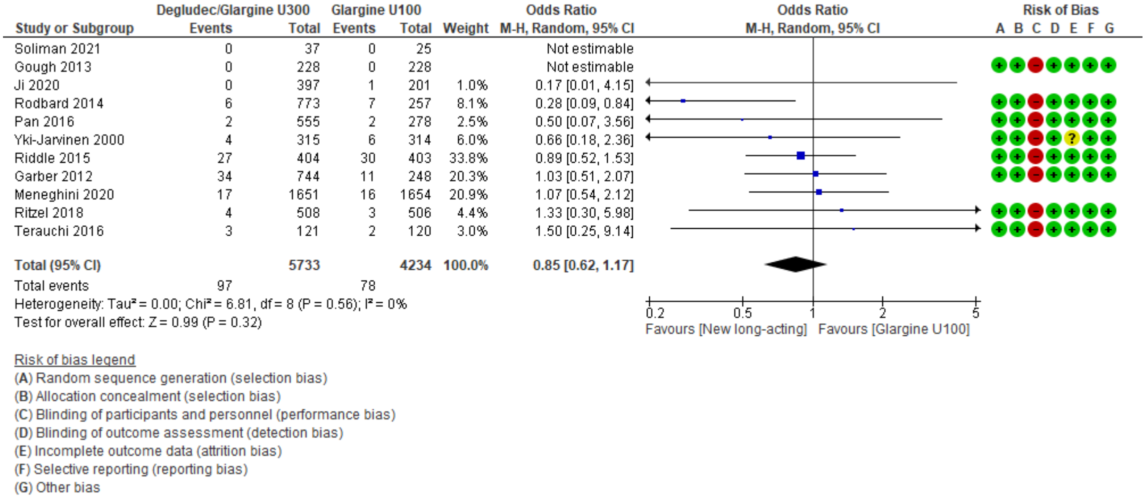

## Grade of evidence

**Table 6**

| Certainty assessment | | | | | | Summary of findings | | | |
|---|---|---|---|---|---|---|---|---|---|
| **Participants (studies) Follow up** | **Risk of bias** | **Inconsistency** | **Indirectness** | **Imprecision** | **Publication bias** | **Overall certainty of evidence** | **Relative effect** (95%, CI) | **Anticipated absolute effects** | |
| | | | | | | | | **Control** | **Intervention** |
| **Total hypoglycemia** | | | | | | | | | |
| 5919 (9 RCT) | serious[a] | not serious | not serious | not serious | very stron association | ⊕⊕⊕⊕ HIGH | **OR 0.68** (0.53;0.88) | 600 per 1000 | **88 lower per 1000** (from 139 to 37 lower) |
| **Nocturnal hypoglycemia** | | | | | | | | | |
| 7584 (12 RCT) | serious[a] | not serious | not serious | not serious | very stron association | ⊕⊕⊕⊕ HIGH | **OR 0.69** (0.60 a 0.79) | 338 per 1.000 | **77 lower per 1.000** (from 103 to 51 lower) |
| **Severe hypoglycemia** | | | | | | | | | |
| 9967 (11 RCT) | serious[a] | not serious | not serious | not serious | none | ⊕⊕⊕◯ MODERATE | **OR 0.85** (0.62 a 1.17) | 18 per 1.000 | 3 lower per 1.000 (from 7 lower to 3 higher) |

**CI:** Confidence interval; **OR:** Odds Ratio; a. Randomization, allocation, and blinding procedures not adequately reported for the majority of included trials;

## Pharmacoeconomic evaluations

The search for pharmaeconomic studies has been updated without retrieving further studies for any of the questions included. Please see the previous version of the guidelines[1,2].

# REFERENCES

1. Mannucci E, Candido R, Delle Monache L, et al. Italian guidelines for the treatment of type 2 diabetes. Nutrition, metabolism, and cardiovascular diseases : NMCD 2022 (In eng). DOI: 10.1016/j.numecd.2022.01.027.
2. Mannucci E, Candido R, Monache LD, et al. Italian guidelines for the treatment of type 2 diabetes. Acta Diabetol 2022 (In eng). DOI: 10.1007/s00592-022-01857-4.
3. Buse JB, Bode BW, Mertens A, et al. Long-term efficacy and safety of oral semaglutide and the effect of switching from sitagliptin to oral semaglutide in patients with type 2 diabetes: a 52-week, randomized, open-label extension of the PIONEER 7 trial. BMJ Open Diabetes Res Care 2020;8(2) (In eng). DOI: 10.1136/bmjdrc-2020-001649.
4. Weinstock RS, Guerci B, Umpierrez G, Nauck MA, Skrivanek Z, Milicevic Z. Safety and efficacy of once-weekly dulaglutide versus sitagliptin after 2 years in metformin-treated patients with type 2 diabetes (AWARD-5): a randomized, phase III study. Diabetes, obesity & metabolism 2015;17(9):849-58. (In eng). DOI: 10.1111/dom.12479.
5. Pfützner A, Paz-Pacheco E, Allen E, Frederich R, Chen R. Initial combination therapy with saxagliptin and metformin provides sustained glycaemic control and is well tolerated for up to 76 weeks. Diabetes, obesity & metabolism 2011;13(6):567-76. (In eng). DOI: 10.1111/j.1463-1326.2011.01385.x.
6. Khaloo P, Asadi Komeleh S, Alemi H, et al. Sitagliptin vs. pioglitazone as add-on treatments in patients with uncontrolled type 2 diabetes on the maximal dose of metformin plus sulfonylurea. J Endocrinol Invest 2019;42(7):851-857. (In eng). DOI: 10.1007/s40618-018-0991-0.
7. Handelsman Y, Lauring B, Gantz I, et al. A randomized, double-blind, non-inferiority trial evaluating the efficacy and safety of omarigliptin, a once-weekly DPP-4 inhibitor, or glimepiride in patients with type 2 diabetes inadequately controlled on metformin monotherapy. Curr Med Res Opin 2017;33(10):1861-1868. (In eng). DOI: 10.1080/03007995.2017.1335638.
8. Terauchi Y, Yamada Y, Ishida H, et al. Efficacy and safety of sitagliptin as compared with glimepiride in Japanese patients with type 2 diabetes mellitus aged >/= 60 years (START-J trial). Diabetes, obesity & metabolism 2017;19(8):1188-1192. (In eng). DOI: 10.1111/dom.12933.
9. Jabbour SA, Frías JP, Ahmed A, et al. Efficacy and Safety Over 2 Years of Exenatide Plus Dapagliflozin in the DURATION-8 Study: A Multicenter, Double-Blind, Phase 3, Randomized Controlled Trial. Diabetes Care 2020;43(10):2528-2536. (In eng). DOI: 10.2337/dc19-1350.
10. Lingvay I, Catarig AM, Frias JP, et al. Efficacy and safety of once-weekly semaglutide versus daily canagliflozin as add-on to metformin in patients with type 2 diabetes (SUSTAIN 8): a double-blind, phase 3b, randomised controlled trial. Lancet Diabetes Endocrinol 2019;7(11):834-844. (In eng). DOI: 10.1016/s2213-8587(19)30311-0.
11. Rodbard HW, Rosenstock J, Canani LH, et al. Oral Semaglutide Versus Empagliflozin in Patients With Type 2 Diabetes Uncontrolled on Metformin: The PIONEER 2 Trial. Diabetes Care 2019;42(12):2272-2281. (In eng). DOI: 10.2337/dc19-0883.
12. Ridderstrale M, Rosenstock J, Andersen KR, Woerle HJ, Salsali A, investigators E-RHHSt. Empagliflozin compared with glimepiride in metformin-treated patients with type 2 diabetes: 208-week data from a masked randomized controlled trial. Diabetes, obesity & metabolism 2018;20(12):2768-2777. DOI: 10.1111/dom.13457.
13. Kaku K, Haneda M, Tanaka Y, et al. Linagliptin as add-on to empagliflozin in a fixed-dose combination in Japanese patients with type 2 diabetes: Glycaemic efficacy and safety profile in a two-part, randomized, placebo-controlled trial. Diabetes, obesity & metabolism 2019;21(1):136-145. DOI: 10.1111/dom.13496.
14. Wang W, Nevárez L, Filippova E, et al. Efficacy and safety of once-weekly dulaglutide versus insulin glargine in mainly Asian patients with type 2 diabetes mellitus on metformin and/or a sulphonylurea: A 52-week open-label, randomized phase III trial. Diabetes, obesity & metabolism 2019;21(2):234-243. (In eng). DOI: 10.1111/dom.13506.

15. Zhang J, Xian TZ, Wu MX, Li C, Pan Q, Guo LX. Comparison of the effects of twice-daily exenatide and insulin on carotid intima-media thickness in type 2 diabetes mellitus patients: a 52-week randomized, open-label, controlled trial. Cardiovasc Diabetol 2020;19(1):48. (In eng). DOI: 10.1186/s12933-020-01014-7.
16. Mannucci E, Naletto L, Vaccaro G, et al. Efficacy and safety of glucose-lowering agents in patients with type 2 diabetes: A network meta-analysis of randomized, active comparator-controlled trials. Nutrition, metabolism, and cardiovascular diseases : NMCD 2021;31(4):1027-1034. (In eng). DOI: 10.1016/j.numecd.2020.12.030.
